# SETDB1 Methylates MCT1 Promoting Tumor Progression by Enhancing the Lactate Shuttle

**DOI:** 10.1002/advs.202301871

**Published:** 2023-08-04

**Authors:** Xiaowei She, Qi Wu, Zejun Rao, Da Song, Changsheng Huang, Shengjie Feng, Anyi Liu, Lang Liu, Kairui Wan, Xun Li, Chengxin Yu, Cheng Qiu, Xuelai Luo, Junbo Hu, Guihua Wang, Feng Xu, Li Sun

**Affiliations:** ^1^ GI Cancer Research Institute Tongji Hospital Huazhong University of Science and Technology Wuhan 430030 P. R. China; ^2^ Tongji Medical College and State Key Laboratory for Diagnosis and Treatment of Severe Zoonotic Infectious Disease Huazhong University of Science and Technology Wuhan Hubei 430030 P. R. China; ^3^ Department of Oncology Tongji Hospital Huazhong University of Science and Technology Wuhan 430030 P. R. China

**Keywords:** lactate shuttle, MCT1 methylation, SETDB1, tumor progression

## Abstract

MCT1 is a critical protein found in monocarboxylate transporters that plays a significant role in regulating the lactate shuttle. However, the post‐transcriptional modifications that regulate MCT1 are not clearly identified. In this study, it is reported that SETDB1 interacts with MCT1, leading to its stabilization. These findings reveal a novel post‐translational modification of MCT1, in which SETDB1 methylation occurs at K473 in vitro and in vivo. This methylation inhibits the interaction between MCT1 and Tollip, which blocks Tollip‐mediated autophagic degradation of MCT1. Furthermore, MCT1 K473 tri‐methylation promotes tumor glycolysis and M2‐like polarization of tumor‐associated macrophages in colorectal cancer (CRC), which enhances the lactate shuttle. In clinical studies, MCT1 K473 tri‐methylation is found to be upregulated and positively correlated with tumor progression and overall survival in CRC. This discovery suggests that SETDB1‐mediated tri‐methylation at K473 is a vital regulatory mechanism for lactate shuttle and tumor progression. Additionally, MCT1 K473 methylation may be a potential prognostic biomarker and promising therapeutic target for CRC.

## Graphical abstract

This discovery suggests that SETDB1‐mediated tri‐methylation at K473 is a vital regulatory mechanism for lactate shuttle and tumor progression. Additionally, MCT1 K473 methylation may be a potential prognostic biomarker and promising therapeutic target for colorectal cancer.

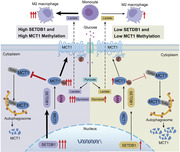

## 1 Introduction

Aerobic glycolysis, also known as the Warburg effect, is a metabolic hallmark of most cancer cells.^[^ ^1^, ^2^, ^3^ ^]^ It fulfills uncontrolled growth's biosynthetic and bioenergetic demands of cancer cells and is accompanied by high lactate generation. In the past, lactate was considered a metabolic waste product of glucose metabolism.^[^ ^4^ ^]^ However, recent studies have revealed the pivotal role of lactate in tumors. Lactate acts as not only a fuel for cancer cells but also a “lactormone” that promotes M2‐like polarization of tumor‐associated macrophages (TAMs) and inhibits the activation of T cells and NK cells, which are based on the lactate shuttle in the tumor microenvironment (TME).^[^ ^5^, ^6^, ^7^ ^]^ Consequently, targeting the lactate shuttle remains an attractive therapeutic intervention for tumors.

Monocarboxylate transporters (MCTs), which belong to the SLC16 gene family, manipulate the lactate shuttle.^[^ ^8^ ^]^ MCT1 and MCT4 are the main mediators. As the core protein of the lactate transmembrane transport channel, MCT1 directly removes intracellular lactate from tumor cells to maintain continuous glycolysis and leads to the accumulation of lactate in the TME.^[^ ^9^ ^]^ Mounting evidence has implicated MCT1 in a plethora of tumor biological functions, including proliferation, metastasis, angiogenesis, metabolism, and immunosuppression.^[^ ^10^, ^11^, ^12^, ^13^, ^14^ ^]^ It has emerged as a potential target for cancer therapy. However, the intrinsic molecular mechanisms dominating MCT1 expression and activity remain to be understood.

Post‐translational modifications (PTMs) enrich the functional diversity of proteins and affect many biological processes in both eukaryotes and prokaryotes.^[^ ^15^, ^16^ ^]^ Site‐specific methylation of non‐histone lysine residues is a prevalent PTM and has been regarded as a novel regulatory mechanism to control protein function, primarily affecting protein stability.^[^ ^17^, ^18^ ^]^ SETDB1, also known as ESET or KMT1E, is a member of the SET domain‐containing histone methyltransferases, and can catalyze H3K9 di‐ and tri‐methylation to repress gene transcription.^[^ ^19^ ^]^ Recently, many studies have highlighted the role of SETDB1‐mediated methylation in non‐histone proteins.^[^ ^20^, ^21^, ^22^ ^]^ For instance, SETDB1 catalyzes p53K370 di‐methylation and promotes its ubiquitination‐mediated degradation, leading to tumor growth.^[^ ^20^ ^]^ Our previous study reveals that SETDB1‐mediated Akt K64 methylation plays a critical role in tumorigenesis.^[^ ^21^ ^]^

In our study, we show that SETDB1 directly interacts with MCT1 and stabilizes it through the direct methylation of the MCT1 protein. Mechanistically, we demonstrate that SETDB1 methylates MCT1 at lysine 473 (K473), which inhibits the interaction between MCT1 and Tollip and blocks Tollip‐mediated autophagic degradation of MCT1. Biologically, we prove that MCT1 K473 methylation promotes tumor glycolysis and M2‐like polarization of TAMs by enhancing lactate export. Thus, our study reveals that MCT1 K473 methylation plays a crucial role in tumor progression and might act as a potential prognostic biomarker and promising therapeutic target for colorectal cancer (CRC).

## 2 Results

### 2.1 SETDB1 Interacts with MCT1 and Enhances Its Expression

**Figure 1:**
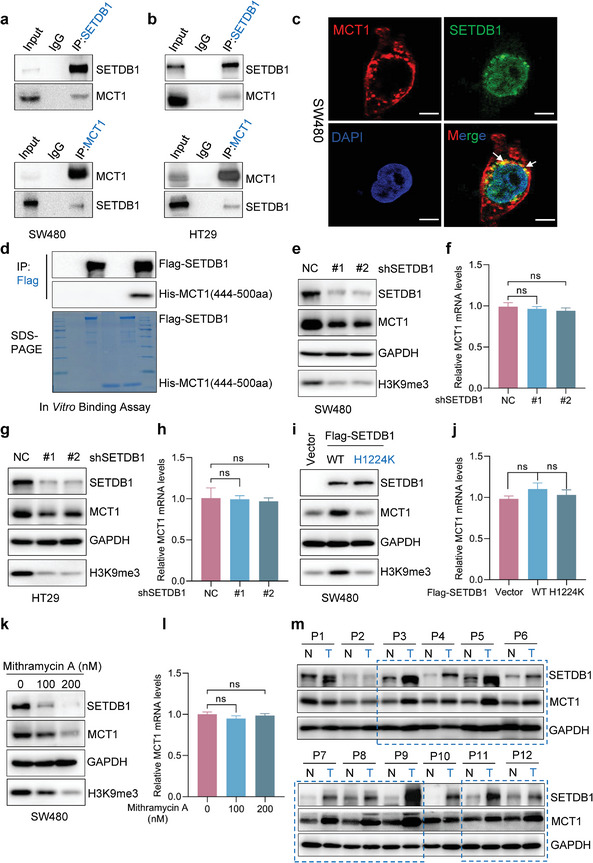
SETDB1 interacts with MCT1 and enhances its expression. a,b) Whole cell lysates (WCL) of SW480 and HT29 cells were collected for IP with anti‐MCT1 or anti‐SETDB1 antibody, followed by immunoblots (IB) analysis. c) The co‐localization of MCT1 and SETDB1 was identified by IF analysis using anti‐MCT1 and anti‐SETDB1 antibodies in SW480 cells. White scale bars, 5 µm. d) In vitro binding assay was performed. Purified Flag‐SETDB1 was incubated with His‐MCT1(444‐500aa) and pulled down using anti‐Flag beads, followed by IB analysis. e–h) Protein and mRNA expression of MCT1 were detected by IB assays and qRT‐PCR assays in SW480 and HT29 cells silenced with control (shNC) or SETDB1 shRNA (#1 and #2). i,j) Protein and mRNA expression of MCT1 were measured by IB assays and qRT‐PCR assays in SW480 cells transfected with Vector, Flag‐SETDB1 (WT), and Flag‐SETDB1 (H1224K) plasmids. k,l) Protein and mRNA expression of MCT1 were determined by IB assays and qRT‐PCR assays in SW480 cells treated with Mithramycin A at the indicated concentrations. m) Colorectal tumors and paired normal tissues were extracted and subjected to detection of MCT1 and SETDB1 protein expression by IB analysis. All immunoblots are performed three times, independently, with similar results. f,h,j,l) Data are represented as mean ± s.d. ns means no significant, by one‐way analysis of variance (ANOVA) with Tukey's test.

In order to investigate the regulatory networks governed by MCT1, we conducted co‐immunoprecipitation (co‐IP) assays to purify MCT1 protein from HEK293T cells overexpressing HA‐MCT1, followed by a systematic mass spectrometry analysis to identify potential MCT1 interacting proteins. One of the candidates MCT1 interacting proteins identified through this analysis was SETDB1 (Figure S1a and Table S1, Supporting Information), a member of the SET domain‐containing histone methyltransferase family that catalyzes tri‐methylation of lysine 9 of histone H3.^[^ ^23^ ^]^ To validate the mass spectrometry results, the interaction between endogenous MCT1 and SETDB1 was confirmed using anti‐MCT1 or anti‐SETDB1 antibodies to carry out co‐IP assays (**Figure** **1**a,b). Furthermore, immunofluorescent co‐localization of MCT1 and SETDB1 was observed through confocal microscopy (Figure 1c). To identify which region of MCT1 interacts with SETDB1, several truncated forms of MCT1 were utilized. Full‐length MCT1 interacted well with SETDB1 and analysis of mutants revealed that the C‐terminal intracellular domain (amino acids 444–500) was required for the interaction between MCT1 and SETDB1 (Figure S1b, Supporting Information). In vitro Flag‐pull down assay further delineated that MCT1 444–500aa directly interacted with SETDB1 (Figure 1d).

We also investigated the functions of the interaction between MCT1 and SETDB1. Firstly, stable knockdown of SETDB1 by short hairpin (sh)RNA in CRC cells resulted in a reduction of endogenous MCT1, while changes in the transcript level of MCT1 were trivial (Figure 1e–h). To further explore whether SETDB1 increased MCT1 protein expression, we transfected different SETDB1 overexpression plasmids into CRC cells and found that only overexpression of wild‐type (WT) SETDB1, but not the SETDB1 H1224K mutant deficient for methyltransferase activity,^[^ ^24^ ^]^ significantly enhanced the expression of MCT1 protein, whereas changes in MCT1 mRNA were insignificant (Figure 1i,j and Figure S1c,d, Supporting Information). A previous study reported that Mithramycin A could impair the activity of the SETDB1 promoter as an inhibitor of SETDB1.^[^ ^25^ ^]^ Consistently, we examined the effect of Mithramycin A treatment in dose‐dependent manners, indicating that not only were the protein levels of SETDB1 and H3K9me3 downregulated, but also the expression of MCT1 was significantly reduced (Figure 1k,l and Figure S1e,f, Supporting Information). Furthermore, we detected the expression of MCT1 in the cytoplasm and cell membrane after treatment with Mithramycin A and found that the protein level of MCT1 in the cytoplasm and cell membrane was decreased (Figure S1g, Supporting Information). Meanwhile, we tested the correlation between the expression of MCT1 and SETDB1 in TCGA database of COAD and READ and found that the Pearson's correlation of the two genes was not statistically significant (Figure S1h, Supporting Information). Subsequently, we conducted an analysis of SETDB1 and MCT1 expression in fresh CRC specimens. Our findings revealed that both SETDB1 and MCT1 were significantly overexpressed in the majority of paired CRC tumor tissues when compared to adjacent normal tissues. Furthermore, we observed a strong correlation between the expression levels of SETDB1 and MCT1 in these CRC specimens. These results indicate a potential association between SETDB1 and MCT1 in CRC progression and support our hypothesis (Figure 1m). Taken together, these findings suggest that SETDB1 directly interacts with MCT1 and enhances the expression of MCT1 at the protein level, without affecting its mRNA expression.

### 2.2 SETDB1 Inhibits the Autophagic Degradation of MCT1

**Figure 2:**
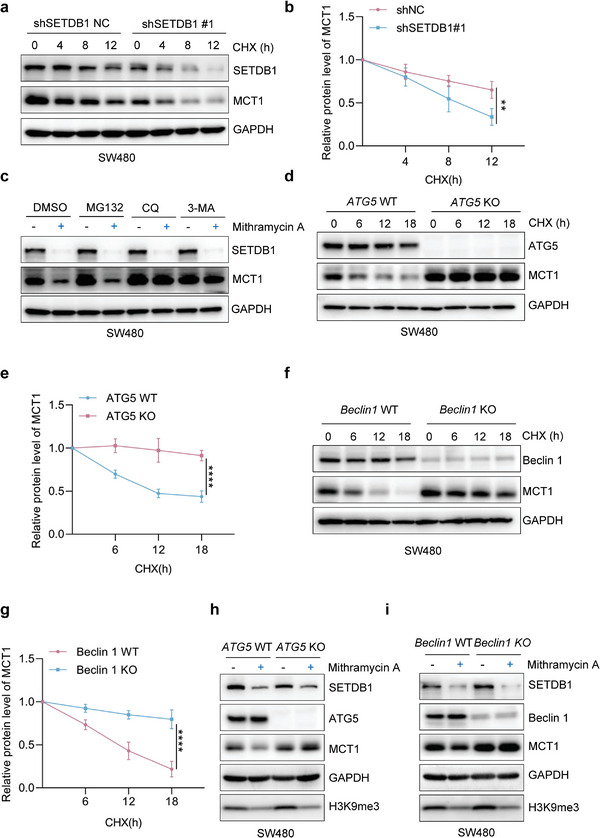
SETDB1 represses the autophagic degradation of MCT1. a) The degradation of MCT1 was detected in shSETDB1NC and shSETDB1#1 SW480 cells treated with CHX (100 µg mL^−1^) for 0, 4, 8, or 12 h by CHX‐chase assay. b) Quantification of the relative protein level of MCT1 in (a). c) The protein level of MCT1 was examined in SW480 cells in the presence of different inhibitors MG132 (10 × 10^−6^ m), CQ (50 × 10^−6^ m), or 3‐MA (5 × 10^−3^ m). d) The degradation of MCT1 was evaluated by CHX‐chase assay in *ATG5* WT or *ATG5* KO SW480 cells. e) Quantification of the relative protein level of MCT1 in (d). f) The degradation of MCT1 was evaluated by CHX‐chase assay in *Beclin 1* WT or *Beclin 1* KO SW480 cells. g) Quantification of the relative protein level of MCT1 in (f). h,i) The protein level of MCT1 in WT and *ATG5* or *Beclin 1* KO SW480 cells treated with dimethyl sulfoxide or Mithramycin A (100 × 10^−^^9^ m, 24 h). All immunoblots were performed three times, independently, with similar results. b,e,g) Data are represented as mean ± s.d. **p < 0.01, ****p < 0.0001, by two‐way ANOVA with Tukey's test.

Our findings suggested that SETDB1 could increase the expression of MCT1, and we aimed to investigate if it could extend the half‐life of MCT1. We treated CRC cells with cycloheximide (CHX) to block translation and observed that knockdown of SETDB1 significantly accelerated the degradation of MCT1 (**Figure** **2**a,b and Figure S2a,b, Supporting Information). Eukaryotic cells utilize the ubiquitin‐proteasome system and the autophagy‐lysosomal pathway as the two primary pathways for protein degradation.^[^ ^26^, ^27^ ^]^ To determine which degradation pathway of MCT1 is predominantly repressed by SETDB1, we treated CRC cells with inhibitors of proteasome or autophagy degradation pathways to detect the degradation of MCT1 under Mithramycin A treatment. Interestingly, we found that inhibiting the expression of SETDB1 could accelerate the autophagic degradation of MCT1 in CRC cells, as indicated by the rescue of MCT1 degradation by the autophagic‐sequestration inhibitor 3‐methyladenine (3‐MA) or the lysosomal‐acidification inhibitor chloroquine (CQ), but not the proteasome inhibitor MG132 (Figure 2c). We further observed that Mithramycin A facilitated Earle's balanced salt solution (EBSS)‐induced MCT1 degradation, and this degradation was significantly regulated by EBSS treatment in a time‐dependent manner (Figure S2c, Supporting Information). We generated *ATG5* or *Beclin1* knockout (KO) cell lines by CRISPR‐Cas9 genome editing (Figure S2d, Supporting Information), in which autophagy was disabled, to confirm whether MCT1 underwent autophagic degradation. Consistently, we found that the degradation of MCT1 was significantly reduced by treating with CHX in *ATG5* or *Beclin 1* KO cell lines (Figure 2d–g). Furthermore, when treating with Mithramycin A in WT and *Beclin 1* or *ATG5* KO cells, the results suggested that Mithramycin A failed to impair the expression of MCT1 under autophagy deficiency (Figure 2h,i). LC3 serves as a reliable indicator of autophagy progression and is widely utilized as a marker for autophagosome membranes. In our study, we observed that the silencing of SETDB1 promoted the binding between LC3 and MCT1, whereas the overexpression of SETDB1 hindered this interaction. These findings suggest that SETDB1 functions to impede the entry of MCT1 into autophagosomes, thereby modulating autophagy processes (Figure S2e,f, Supporting Information). These data collectively indicate that SETDB1 stabilizes MCT1 by inhibiting its autophagic degradation.

### 2.3 SETDB1 Induces Tri‐Methylation of Lysine 473 on MCT1

We aimed to investigate the precise molecular mechanism behind the SETDB1‐mediated stabilization of MCT1. Recent studies have highlighted the role of PTMs in regulating protein stability, such as phosphorylation, acetylation, ubiquitination, and methylation.^[^ ^28^ ^]^ As an H3K9 methyltransferase, SETDB1 can methylate non‐histone proteins, including P53 and AKT.^[^ ^20^, ^21^, ^22^ ^]^ Notably, SETDB1 can form a complex with P53 and catalyze P53K370 di‐methylation, which promotes P53 degradation by MDM2.^[^ ^20^ ^]^ Based on our above results, we hypothesized that SETDB1 regulated MCT1 expression via MCT1 methylation.

**Figure 3:**
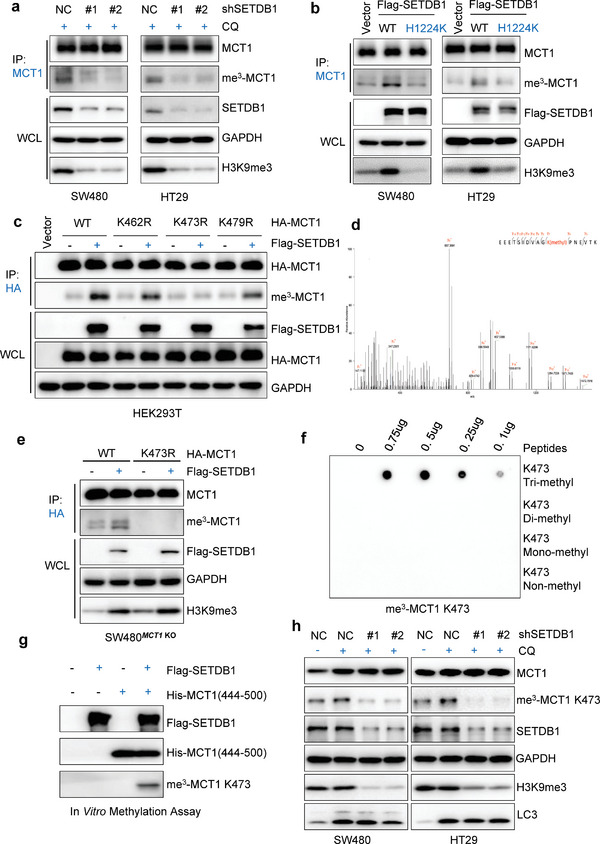
SETDB1 induces tri‐methylation of lysine 473 on MCT1. a) WCL collected from SW480 and HT29 cells silenced with control (ShNC) or SETDB1 ShRNA (#1, #2) were subjected to IP assay with anti‐MCT1 antibody, followed by IB analysis. b) WCL collected from SW480 and HT29 cells transfected with Vector, Flag‐SETDB1 (WT), and Flag‐SETDB1 (H1224K) plasmids were subjected to IP assay with anti‐MCT1 antibody, followed by IB analysis. c) HEK293T cells transfected with HA‐MCT1 WT or mutant plasmids as indicated, then transfected with Vector or Flag‐SETDB1, WCL were collected for IP with anti‐HA beads, followed by IB analysis. d) Secondary mass spectrometry result of lysine 473 methylation residue. e) SW480*^MCT1^* ^KO^ cells transfected with HA‐MCT1 WT or K473R plasmid as indicated, then transfected with Vector or Flag‐SETDB1, WCL were collected for IP with anti‐HA beads, followed by IB analysis. f) Different peptides were added into PVDF membranes at indicated concentrations, followed by a dot blot assay using MCT1 K473 specific tri‐methylation antibody. g) In vitro methylation assay was performed. Purified Flag‐SETDB1 was incubated with His‐MCT1(444‐500aa) in the presence of S‐adenosyl‐L‐methionine, followed by IB analysis to analyze MCT1 methylation using MCT1 K473‐specific tri‐methylation antibody. h) WCL collected from SW480 and HT29 cells silenced with control (ShNC) or SETDB1 ShRNA (#1, #2) were subjected to IB assay with MCT1 K473‐specific tri‐methylation antibody. All immunoblots were performed three times, independently, with similar results.

To test this hypothesis, we transfected HA‐MCT1 into HEK293T cells and found that SETDB1 overexpression increased tri‐methylation of MCT1 but not mono‐ or di‐methylation (Figure S3a, Supporting Information). Because MCT4 is highly conserved with MCT1, we transfected HA‐MCT4 into HEK293T cells and found that SETDB1 overexpression did not increase mono‐, di‐, and tri‐methylation of MCT4 (Figure S3a, Supporting Information). Subsequently, we performed a co‐IP assay to analyze the endogenous MCT1 methylation in CRC cells, and the results showed that downregulating SETDB1 or treatment with Mithramycin A led to a significant decrease in MCT1 tri‐methylation (**Figure** **3**a and Figure S3b, Supporting Information). Overexpression of WT SETDB1, but not the SETDB1 H1224K mutant, also significantly increased MCT1 tri‐methylation (Figure 3b), suggesting that MCT1 is a methylated substrate of SETDB1.

Since SETDB1 is an N‐methyltransferase, we sought to identify the potential SETDB1‐dependent MCT1 methylation site. We performed a co‐IP assay to enrich MCT1 protein from HEK293T cells overexpressing HA‐MCT1 and performed mass spectrometry analysis to identify the lysine methylation site. The results identified three potential lysine residues (lysine 462, 473, and 479) located on the C‐terminal intracellular domain of MCT1 (Figure S3c and Table S2, Supporting Information). Next, we constructed MCT1 plasmids with variants of the three lysine sites deficient in methylation and transfected them into HEK293T cells with or without SETDB1 overexpression. The results showed that only MCT1 K473 tri‐methylation upregulation depended on SETDB1 (Figure 3c). Importantly, the K473 methylation site on MCT1 was conserved in mammals and confirmed by a second mass spectrometry analysis (Figure S3d, Supporting Information and Figure 3d).

We generated *MCT1* KO CRC cell lines using the CRISPR‐Cas9 system and stably expressed a methylation‐deficient variant of MCT1 K473R in MCT1 knockout SW480 cells (Figure S3e,f, Supporting Information). Consistent with the data, the methylation‐deficient variant of MCT1 K473R abolished tri‐methylation upregulation of MCT1 in CRC cells (Figure 3e). We also generated an antibody that specifically recognized K473 tri‐methylation and verified it by dot blot analysis (Figure 3f). Furthermore, we performed immunohistochemistry (IHC) analysis on subcutaneous tumor tissue obtained from MCT1 wild‐type (WT) and MCT1 K473 SW480 cells. This analysis provided additional confirmation that the MCT1 K473 tri‐methylation antibody specifically recognized MCT1 K473 tri‐methylation (Figure S3g, Supporting Information). An in vitro methylation assay confirmed that SETDB1 could methylate MCT1 at K473 (Figure 3g). Using the specific antibody, we observed that SETDB1 knockdown significantly impaired MCT1 K473 tri‐methylation, while overexpression of WT SETDB1 but not the SETDB1 H1224K mutant enhanced MCT1 K473 tri‐methylation (Figure 3h and Figure S3h, Supporting Information). In addition, we observed that the K473 methylation of MCT1 was completely abolished in K473R cells compared to WT MCT1 cells (Figure S3i, Supporting Information). Taken together, our data provide strong evidence to support our hypothesis that SETDB1 induces tri‐methylation of MCT1 at K473, which in turn inhibits the degradation of MCT1.

### 2.4 Methylation of MCT1 at K473 Tri‐Methylation Hinders Tollip‐Mediated Autophagic Degradation of MCT1

**Figure 4:**
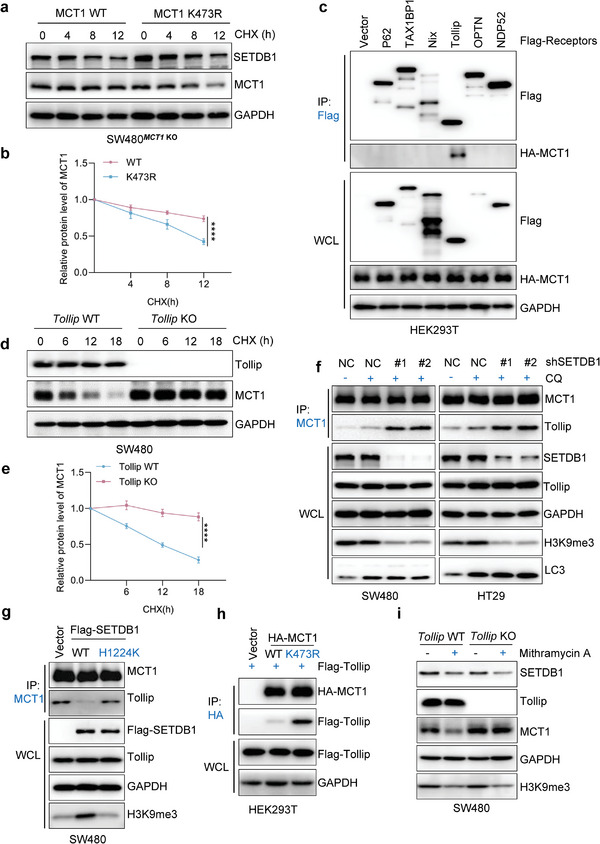
MCT1 K473 tri‐methylation blocks Tollip‐mediated autophagic degradation of MCT1. a) The degradation of MCT1 was detected in SW480*^MCT1^* ^KO^ cells stably expressing MCT1 WT or MCT1 K473R cells treated with CHX (100 µg mL^−1^) for 0, 4, 8, or 12 h by CHX‐chase assay. b) Quantification of relative protein level of MCT1 in (a). c) HEK293T cells were transfected with HA‐MCT1 and indicated Flag‐tagged cargo receptors, WCL were collected for IP with anti‐Flag beads, followed by IB analysis. d) The degradation of MCT1 was evaluated by CHX‐chase assay in *Tollip* WT or *Tollip* KO SW480 cells. e) Quantification of the relative protein level of MCT1 in (d). f) WCL collected from SETDB1 knockdown SW480 and HT29 cells were subjected to IP assay with anti‐MCT1 antibody, followed by IB analysis. g) WCL collected from SW480 cells transfected with Vector, Flag‐SETDB1 (WT), and Flag‐SETDB1 (H1224K) plasmids were subjected to IP assay with anti‐MCT1 antibody, followed by IB analysis. h) HEK293T cells were co‐transfected with Flag‐Tollip and Vector, HA‐MCT1 WT or HA‐MCT1 K473R, and WCL was collected for IP with anti‐HA beads, followed by IB analysis. i) The protein level of MCT1 in *Tollip* WT and *Tollip* KO SW480 cells treated with DMSO or Mithramycin A (100 × 10^−9^ m, 24 h). All immunoblots were performed three times, independently, with similar results. a,b) Data are represented as mean ± s.d. ****p < 0.0001, by two‐way ANOVA with Tukey's test.

We next investigated the half‐life of MCT1 WT and MCT1 K473R and found that the latter had a much shorter half‐life (**Figure** **4**a,b), consistent with our previous conclusions. We further demonstrated that only 3‐MA or CQ, but not MG132, rescued the accelerated degradation of the methylation‐deficient mutant MCT1 K473, indicating that MCT1 K473 methylation could decelerate the autophagic degradation of MCT1 in CRC cells (Figure S4a,b, Supporting Information). Additionally, we examined the ubiquitination level of WT and methylation‐deficient MCT1 and found no difference (Figure S4c, Supporting Information). Since cargo receptors are crucial for delivering substrates for selective autophagic degradation,^[^ ^29^ ^]^ we investigated which cargo receptor is responsible for the autophagic degradation of MCT1. Our co‐IP assays showed that MCT1 mainly interacted with Tollip among various cargo receptors (Figure 4c). We then constructed *Tollip* KO CRC cell lines using the CRISPR‐Cas9 system and found that the degradation of MCT1 was significantly reduced by CHX treatment in *Tollip* KO cell lines (Figure S4d, Supporting Information and Figure 4d,e), suggesting that cargo receptor Tollip mediates the selective autophagic degradation of MCT1.

Since lysine methylation can alter the interactions between proteins, we explored whether MCT1 K473 methylation could affect the interaction between MCT1 and Tollip. Our results showed that silencing SETDB1 facilitated Tollip binding with MCT1 while overexpressing SETDB1 restrained the interaction (Figure 4f,g). Moreover, co‐IP assays demonstrated that methylation‐deficient mutant MCT1 K473 could interact with more Tollip protein compared to WT MCT1 (Figure 4h and Figure S4e, Supporting Information). Confocal microscopy further demonstrated that SETDB1 overexpression dramatically reduced the colocalization of MCT1 and Tollip, while Mithramycin A treatment significantly enhanced SETDB1‐MCT1 colocalization (Figure S4f,g, Supporting Information). Furthermore, treating *Tollip* WT and *Tollip* KO cells with Mithramycin A failed to impair the expression of MCT1 under Tollip deficiency (Figure 4i). Collectively, these results suggest that SETDB1‐mediated MCT1‐K473 tri‐methylation attenuates the binding of MCT1 and Tollip, preventing MCT1 delivery to the autophagosome for selective degradation.

### 2.5 MCT1 K473 Tri‐Methylation Promotes both Tumor Glycolysis and M2‐Like Polarization of TAMs by Regulating the Transport of Lactate

**Figure 5:**
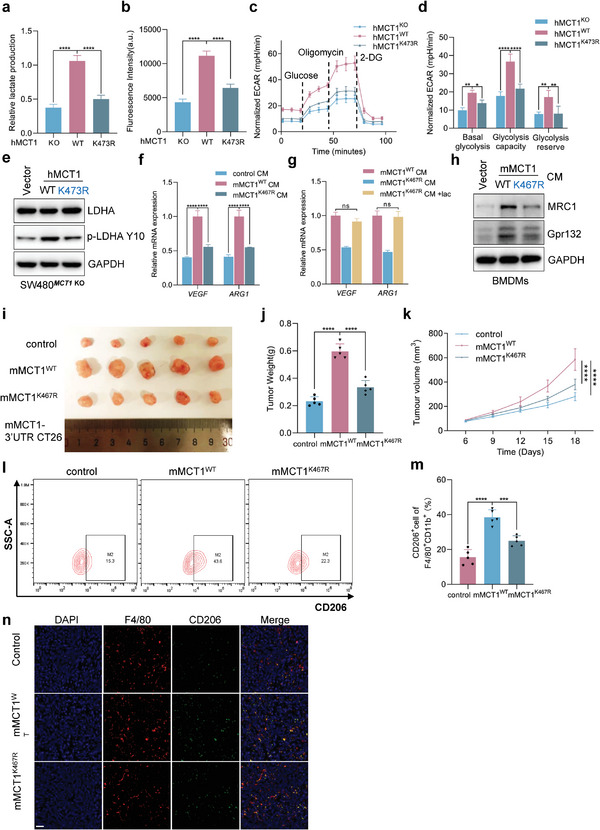
MCT1 K473 tri‐methylation promotes tumor glycolysis and M2‐like polarization of TAMs by regulating lactate transport. a) Lactate production was measured in the conditioned medium (CM) of SW480*^MCT1^* ^KO^ cells stably expressing Vector, MCT1 WT, or MCT1 K473R, *n* = 3. b) Glucose uptake was measured in SW480*^MCT1^* ^KO^ cells stably expressing Vector, MCT1 WT, or MCT1 K473R, *n* = 3. c,d) Bioenergetic analysis was performed with the Seahorse XF24 analyzer platform. ECAR of SW480*^MCT1^* ^KO^ cells stably expressing Vector, MCT1 WT, or MCT1 K473R was measured and calculated, *n* = 3. e) WCL collected from SW480*^MCT1^* ^KO^ cells stably expressing Vector, MCT1 WT, or MCT1 K473R, followed by IB assay. f) The relative mRNA levels of *VEGF* and *ARG1* macrophage markers in BMDMs treated with indicated CM, *n* = 3. g) The relative mRNA levels of *VEGF* and *ARG1* macrophage markers in BMDMs treated with indicated CM containing with or without lactate (5 × 10^−3^ m), *n* = 3. h) WCL collected from BMDMs treated by the indicated CM, followed by IB assay. i) A syngeneic tumor model was performed by injecting MCT1‐3′UTR CT26 cells stably expressing Vector, mus‐MCT1 WT, or mus‐MCT1 K467R cells into BALB/c mice. *n* = 5 mice. j,k) Quantification of tumor weight and volume of tumors generated in (i). l,m) Flow cytometry analysis of macrophage polarization of tumors generated in (i). n) Representative images for infiltration of intratumor CD206^+^ and F4/80^+^ cells by IF. White scale bars, 20 µm. All immunoblots were performed three times, independently, with similar results. Data are represented as mean ± s.d. *p < 0.05, **p < 0.01, ***p < 0.001, ****p < 0.0001; ns means no significant, by a,b,j,m) one‐way ANOVA with Tukey's test and d,f,g,k) two‐way ANOVA with Tukey's test.

MCT1, as a lactate transporter, plays a crucial role in regulating cellular metabolism, specifically glycolysis.^[^ ^9^ ^]^ Thus, we initially investigated the effect of MCT1 K473 tri‐methylation on lactate transport. We observed a significant decrease in lactate export in MCT1 K473R CRC cells compared to MCT1 WT cells (**Figure** **5**a), which was consistent with the results of SETDB1 knockdown (Figure S5a,b, Supporting Information). Additionally, MCT1 K473R cells had a much lower glucose uptake ability than MCT1 WT cells, reflecting decreased glycolysis activation, and the same results were observed in cells with SETDB1 konckdown. (Figure 5b and Figure S5c,d, Supporting Information). The glycolytic stress tests further showed that MCT1 K473 methylation deficiency inhibited basal glycolysis, glycolysis capacity, and glycolysis reserve in MCT1 K473R cells (Figure 5c,d). Similarly, knockdown of SETDB1 exhibited weaker basal glycolysis, glycolysis capacity, and glycolysis reserve (Figure S5e,f, Supporting Information). Lactate dehydrogenase (LDHA) is an enzyme that plays a crucial role in converting pyruvate into lactate. It has been observed that LDHA can be phosphorylated at tyrosine 10 (Tyr10) by HER2 and Src, leading to an increase in its enzymatic activity and consequently promoting elevated glycolysis and lactate production.^[^ ^30^ ^]^ Hence, we detected the LDHA and pLDHA(Tyr10) protein level and found that MCT1 K473 methylation deficiency decreased the level of pLDHA(Tyr10) (Figure 5e).These findings demonstrated that MCT1 K473 tri‐methylation enhances lactate export and tumor cell glycolysis.

Furthermore, previous research suggests that tumor‐derived lactate can modulate M2‐like macrophage polarization of TAMs, even though lactate is a by‐product of anaerobic glycolysis.^[^ ^6^, ^31^ ^]^ Consistently, we constructed MCT1 3′UTR knockdown CT26 cell lines, which were rescued with mus‐MCT1 WT and mus‐MCT1 K467R, homologous to human MCT1 K473, and measured lactate export and glucose uptake ability (Figure S5g, Supporting Information). The results were similar to human‐derived tumor cells (Figure S5h–k, Supporting Information). We obtained the <3 kDa fraction containing lactate from a conditioned medium (CM) collected from CRC cells to stimulate bone marrow‐derived macrophage (BMDM) cells. The results showed that the <3 kDa fraction from MCT1 K467R CT26 cells decreased the expression of *VEGF* and *ARG1* in BMDMs, the marker of M2‐like macrophage polarization (Figure 5f). To investigate the impact of lactate levels in the cell culture media (CM) on M2‐like macrophage polarization, we supplemented lactate in the CM derived from MCT1 K467R CT26 cells. Our results, as shown in Figure 5g, revealed that the supplementation of lactate led to a restoration of VEGF and ARG1 expression, indicating that lactate was responsible for the decreased M2‐like macrophage polarization observed in the context of MCT1 methylation deficiency. Furthermore, previous research has demonstrated that lactate can activate macrophage Gpr132, thereby promoting M2‐like macrophage polarization.^[^ ^31^ ^]^ In our study, we observed a significant decrease in Gpr132 protein levels in BMDMs stimulated by the CM from MCT1 K467R CT26 cells compared to those stimulated by the WT CM (Figure 5h). Furthermore, in a subcutaneously implanted tumor model, we observed that deficiency of MCT1 methylation inhibited tumor progression (Figure 5i–k) and decreased the number of CD206^+^TAMs in the primary subcutaneous tumors of MCT1 K467R group compared to MCT1 WT group (Figure 5l,m). Consistently, immunofluorescence (IF) staining of tumors confirmed the similar result with the flow cytometry results (Figure 5n). Moreover, SETDB1 knockdown CT26 cells exhibited a lower tumorigenic potential than control cells and reduced the percentage of CD206^+^TAMs in vivo (Figure S5l‐p, Supporting Information). These findings indicate that MCT1 methylation‐deficient mutant impairs tumor glycolysis and M2‐like polarization of TAMs by inhibiting lactate export.

### 2.6 MCT1 K473 Tri‐Methylation is Required for SETDB1‐Mediated Tumor Glycolysis and M2 Polarization of TAMs

**Figure 6:**
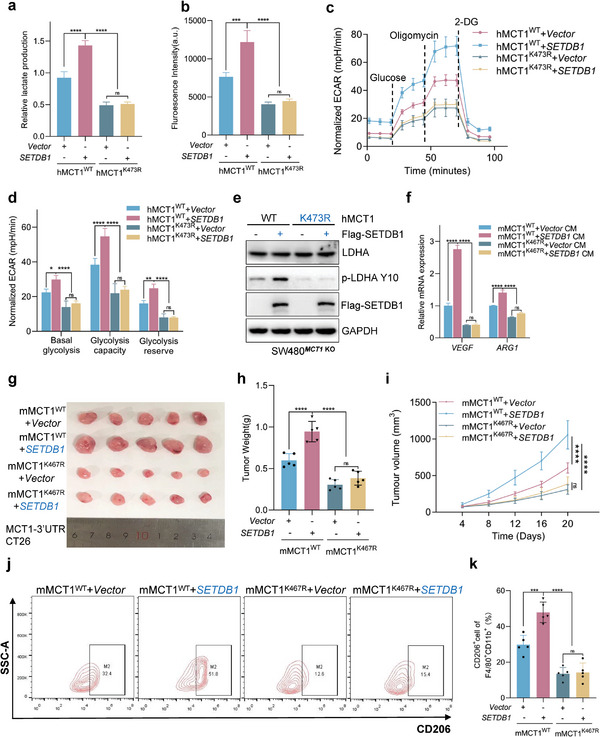
MCT1 K473 tri‐methylation is required for SETDB1‐mediated tumor glycolysis and M2‐like polarization of TAMs. a) Lactate production was measured in the conditioned medium (CM) of SW480*^MCT1^* ^KO^ cells stably expressing MCT1 WT or MCT1 K473R and transfected with Vector or Flag‐SETDB1, *n* = 3. b) Glucose uptake was measured in SW480*^MCT1^* ^KO^ cells stably expressing MCT1 WT or MCT1 K473R and transfected with Vector or Flag‐SETDB1, *n* = 3. c,d) Bioenergetic analysis was performed with the Seahorse XF24 analyzer platform. ECAR of SW480*^MCT1^* ^KO^ cells stably expressing MCT1 WT or MCT1 K473R and transfected with Vector or Flag‐SETDB1 was measured and calculated, *n* = 3. e) WCL collected from SW480*^MCT1^* ^KO^ cells stably expressing MCT1 WT or MCT1 K473R and transfected with Vector or Flag‐SETDB1, followed by IB assay. f) The relative mRNA levels of *VEGF* and *ARG1* macrophage markers in BMDMs were treated with indicated CM, *n* = 3. g) A syngeneic tumor model was performed by injecting MCT1‐3′UTR CT26 cells stably expressing MCT1 WT or MCT1 K473R cells and overexpression Vector or SETDB1 into BALB/c mice. *n* = 5 mice. h,i) Quantification of tumor weight and volume of tumors generated in (g). j,k) Flow cytometry analysis of macrophage polarization of tumors generated in (g). All immunoblots were performed three times, independently, with similar results. Data are represented as mean ± s.d. *p < 0.05, **p < 0.01, ***p < 0.001, ****p < 0.0001; ns means no significant, by a,b,h,k) one‐way ANOVA with Tukey's test and d,f,i) two‐way ANOVA with Tukey's test.

Our findings have demonstrated that both MCT1 K473 tri‐methylation and SETDB1 can promote tumor glycolysis and M2‐like polarization of TAMs. However, the relationship between MCT1 K473 tri‐methylation and SETDB1 in cancer promotion is currently unknown. To investigate this, we stably overexpressed SETDB1 in MCT1 WT and MCT1 K473R CRC cells and conducted lactate production and glucose uptake assays. The results showed that overexpressing SETDB1 increased lactate export and glucose uptake, but only had a partial effect on MCT1 K473R cells (**Figure** **6**a,b). Furthermore, the Seahorse glycolytic stress tests demonstrated that the activation of glycolysis in MCT1 WT cells could be significantly improved by overexpressing SETDB1, unlike that of MCT1 K473R cells (Figure 6c,d). We then conducted WB assay and found that SETDB1‐overexpressing MCT1 WT cells enhanced pLDHA(Tyr10) expression, but not in MCT1 K473R cells (Figure 6e). Similarly, we constructed a stably expressing vector (SETDB1) in MCT1‐3′UTR CT26 cells that stably expressed mus‐MCT1 WT or mus‐MCT1 K467R, and collected the CM to stimulate BMDMs (Figure S6a, Supporting Information). The results revealed that the <3 kDa fraction from SETDB1‐overexpressing MCT1 WT CT26 cells enhanced *VEGF* and *ARG1* mRNA expression and Gpr132 protein levels, but not in MCT1 K467R CT26 cells (Figure 6f and Figure S6b, Supporting Information). Consistent results were also obtained from lactate export and glucose uptake assays using human‐derived tumor cells (Figure S6c,d, Supporting Information). Encouraged by these data, we conducted a subcutaneously implanted tumor model by injecting these cells into BALB/c mice, which showed that SETDB1 could significantly promote tumor progression in MCT1 WT CT26 cells, but not in MCT1 K467R CT26 cells (Figure 6g–i). Flow cytometry analysis of TAMs in implanted tumors showed that SETDB1 significantly increased the percentage of CD206^+^TAMs in MCT1 WT tumors, whereas this effect was not observed in MCT1 K467R tumors (Figure 6j,k). Consistently, IF staining of tumors confirmed the similar result with the flow cytometry results (Figure S6e, Supporting Information). In summary, our data suggest that MCT1 K473 tri‐methylation is necessary for SETDB1‐mediated tumor glycolysis and M2‐like polarization of TAMs.

### 2.7 MCT1 K473 Tri‐Methylation is Positively Related to CRC and has Prognostic Significance in CRC Patients

**Figure 7:**
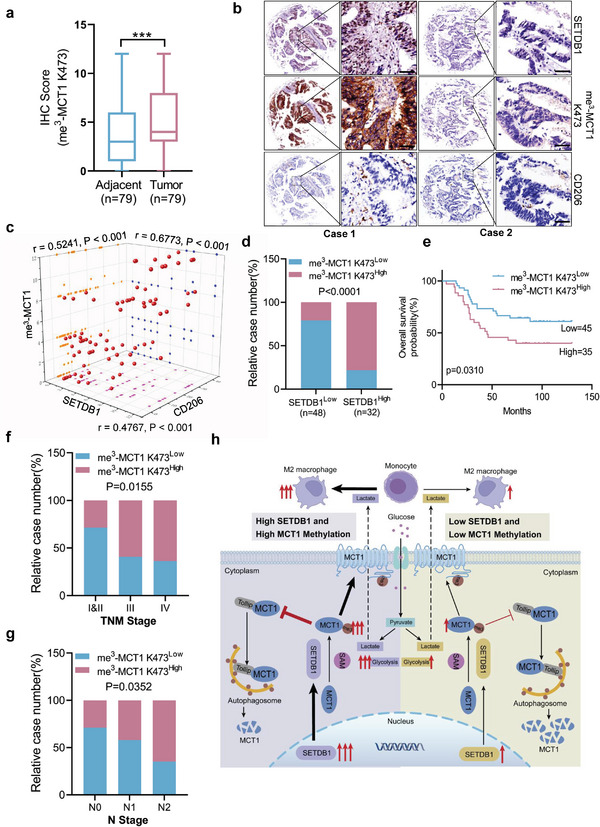
MCT1 K473 tri‐methylation is positively related to CRC and has prognostic significance in CRC patients. a) MCT1 K473 tri‐methylation IHC staining score was detected in tumor and adjacent tissues, *n* = 79. Student's two‐tailed *t*‐test, *p* < 0.001. b) Representative image of IHC staining for SETDB1, MCT1 K473 tri‐methylation, and CD206 in colorectal cancer. Black scale bar, 50 µm . c) Scatter plot of the IHC staining scores for SETDB1, MCT1 K473 tri‐methylation, and CD206 in CRC, *n* = 80. All *p* and *r* values were calculated with Spearman's *r* test. c) Quantitative IHC staining score showing the correlation of SETDB1 and MCT1 K473 tri‐methylation. Chi‐square test, *p* < 0.0001. d) Correlation between SETDB1 and MCT1 K473 tri‐methylation expression was determined by Pearson correlation coefficient test, *p* < 0.0001. e) Kaplan–Meier analysis of overall survival in a set of 80 colorectal cancer patients according to MCT1 K473 tri‐methylation expression. Log‐rank test, *p* = 0.0310. f,g) Quantitative IHC staining score showing the correlation between MCT1 K473 tri‐methylation and TNM stage or N stage using microarray of colorectal cancer specimen. Chi‐square test, *p* = 0.0155 and *p* = 0.0352, respectively. h) The working model of SETDB1‐mediated MCT1 K473 tri‐methylation.

The Cancer Genome Atlas database data indicate that SETDB1 is significantly upregulated in various cancers including colorectal, liver, and gastric cancer. Moreover, Kaplan–Meier analysis demonstrates that high SETDB1 expression is positively correlated with poor survival outcomes in colorectal and gastric cancer patients (Figure S7a–d, Supporting Information). To further explore the relationship between SETDB1, MCT1 K473 tri‐methylation, and M2‐like polarization of TAMs in CRC, we conducted IHC assays on a CRC tissue microarray using anti‐SETDB1, anti‐me3 MCT1 K473, and anti‐CD206 antibodies (Figure S7e, Supporting Information). Our results revealed that MCT1 methylation was expressed at higher levels in CRC tissues compared to adjacent tissues (**Figure** **7**a), and MCT1 K473 tri‐methylation levels positively correlated with SETDB1 protein levels and CD206 protein levels in different specimens (Figure 7b–d). Additionally, we performed IHC assays on a CRC tissue microarray consisting of 30 paired clinical specimens using anti‐SETDB1 antibody. Two of the tissue microarray results demonstrated that SETDB1 expression levels were higher in CRC tissues compared to adjacent tissues (Figure S7f, Supporting Information). Moreover, we found that patients with high SETDB1 and MCT1 methylation expression had a significantly longer overall survival time compared to those with low SETDB1 and MCT1 methylation expression (Figure 7e and Figure S7g, Supporting Information). Additionally, we analyzed the relationship between MCT1 K473 methylation and clinicopathological characteristics in CRC specimens by conducting IHC assays, and the data revealed a positive correlation between the IHC score of MCT1 methylation and TNM stage and N stage in CRC patients (Figure 7f,g). In summary, our results demonstrate that SETDB1‐mediated MCT1 methylation is a critical factor in CRC, and MCT1 K473 tri‐methylation has the potential to be a predictive marker for cancer outcome.

## 3 Discussion

MCT1, also known as SLC16A1, is highly upregulated in various tumor tissues and plays a crucial role in exporting lactate to the TME.^[^ ^9^, ^32^, ^33^ ^]^ However, the post‐transcriptional regulation of MCT1 has been limited to miRNAs,^[^ ^34^, ^35^, ^36^ ^]^ and its PTM has not been fully elucidated. Recent studies have highlighted the significance of PTMs in regulating protein stability, and lysine methylation has been shown to affect the stability of many key non‐histone proteins.^[^ ^17^, ^28^ ^]^ Our study found that SETDB1‐mediated MCT1 methylation at lysine 473 regulates the stability of MCT1 and blocks Tollip‐mediated autophagic degradation of MCT1. This study replenishes the list of PTMs of MCT1 and sheds light on its regulation by methylation.

Lactate plays a critical role in tumor progression as it is involved in the maintenance of continuous aerobic glycolysis and regulating immune cells' function in TME, leading to immune escape and M2‐like macrophage polarization.^[^ ^6^, ^37^, ^38^, ^39^, ^40^ ^]^ Our study revealed that MCT1 methylation increases the protein stability of MCT1, promoting lactate export in CRC. SETDB1‐mediated MCT1 tri‐methylation at K473 enhances cancer cell glycolysis and promotes M2‐like macrophage polarization of TAMs. In addition, M2 macrophages participate in tumor progression through the secretion of cytokines and exosomes.^[^ ^41^, ^42^ ^]^ Our findings suggest that the methylation‐deficient variant of MCT1 inhibits subcutaneous tumor growth, which may be partially attributed to lactate‐mediated macrophage. In conclusion, MCT1 methylation is the central molecular mechanism of MCT1‐mediated lactate shuttle, highlighting its potential as a therapeutic target for CRC.

Protein lysine methylation is catalyzed by protein lysine methyltransferases (PKMTs), and dysregulation of these PKMTs is observed in a variety of human malignancies.^[^ ^43^, ^44^ ^]^ SETDB1 is a methyltransferase enzyme that has the ability to catalyze the methylation of histone H3K9, lysine 370 of p53, and lysine 64, 140, and 142 of Akt.^[^ ^20^, ^21^, ^22^, ^23^ ^]^ Previous research has indicated that SETDB1 plays a critical role in the inactivation of the X chromosome, repression of endogenous retroviruses, regulation of intestinal epithelial differentiation, and suppression of tumor intrinsic immunogenicity.^[^ ^45^, ^46^, ^47^, ^48^ ^]^ Furthermore, it has been established that SETDB1 can exert its repressive function on gene expression through the modification of histone marks, specifically H3K9me2 and H3K9me3.^[^ ^19^ ^]^ Nevertheless, our study revealed that although SETDB1 affected the expression of MCT1, manipulating SETDB1 levels did not have an impact on the transcriptional regulation of MCT1. This suggests that the influence of SETDB1 on MCT1 protein stability was not reliant on the activity of H3K9me2 and H3K9me3. Study showed that SETDB1 is upregulated and has a poor outcome in CRC, which is consistent with previous studies.^[^ ^49^ ^]^ However, it is important to interpret this result cautiously, considering that other factors such as treatment approaches and patient comorbidities may also contribute to patient outcomes.

Notably, our findings indicated that high SETDB1 expression is correlated with MCT1 K473 tri‐methylation in CRC tissues, which is related to poor survival time in CRC patients. This suggests that SETDB1‐mediated methylation of MCT1 is positively correlated with CRC patients’ overall survival time, and MCT1 methylation at K473 might be a potential predictive marker for cancer outcome. We provide a mechanistic elucidation of MCT1 methylation induced by SETDB1, which may help develop a therapeutic peptide to block the methylation of MCT1 K473. Moreover, we will conduct clinical studies to investigate the potential therapeutic implications of targeting MCT1 K473 tri‐methylation and SETDB1 in cancer treatment.

In summary, we have discovered a mechanism involving SETDB1‐mediated MCT1 methylation that leads to the development of CRC. MCT1 is a new substrate of SETDB1 and is stabilized by SETDB1 through inhibiting the interaction between MCT1 and Tollip, thus blocking Tollip‐mediated autophagic degradation of MCT1. Consequently, inhibiting MCT1 K473 methylation impairs the lactate shuttle, restrains tumor glycolysis, and further restrains M2‐like polarization of TAMs. Therefore, our findings reveal a new function for the lysine methylation SETDB1/MCT1 pathway in the regulation of CRC progression, suggesting that SETDB1 may be a potential therapeutic target for CRC.

## 4 Experimental Section

### Cell Lines and Cell Culture

Human embryonic kidney cells HEK293T, human CRC cell lines (SW480 and HT29), and mouse colon cancer cell line CT26 were obtained from American Type Culture Collection. HEK293T, SW480, HT29, and CT26 were, respectively, cultured in Dulbecco's modified Eagle medium, Leibovitz's L‐15 medium, McCoy's 5a medium, and RPMI‐1640 medium with 10% fetal bovine serum, 1% penicillin, and 1% streptomycin. Cells were cultured in a humidified cell‐culture incubator containing 5% CO_2_ at 37 °C.

### Reagents and Antibodies

Mithramycin A (HY‐A0122), MG132 (HY‐13259), chloroquine phosphate (CQ) (HY‐17589), 3‐methyladenine (3‐MA) (HY‐19312), CHX (HY‐12320), anti‐HA magnetic beads (HY‐K0201), anti‐Flag magnetic beads (HY‐K0207), and Protein A/G magnetic beads (HY‐K0202) were purchased from MedChemExpress. EBSS (C0213), puromycin dihydrochloride (ST551), and G418 (ST081) were purchased from Beyotime. The primary antibodies for MCT1 (sc‐365501; Santa Cruz Biotechnology, ab93048; Abcam and A3013; ABclonal), SETDB1 (2196; Cell Signaling Technology and A6145; ABclonal), GAPDH (AC033; ABclonal), Tollip (A21551; ABclonal), ATG5 (10181‐2‐AP; Proteintech), Beclin 1 (11306‐1‐AP; Proteintech), Caveolin‐1 (A19006; ABclonal), Histone H3 (4499; Cell Signaling Technology), trimethyl‐Histone H3‐K9 (A2360; ABclonal), Flag tag (14793S; Cell Signaling Technology), HA tag (51064‐2‐AP; Proteintech), His tag (AE003; ABclonal), Pan mono‐methyl lysine (A18293; ABclonal), Pan di‐methyl lysine (14117; Cell Signaling Technology), Pan tri‐methyl lysine (14680; Cell Signaling Technology), LDHA (A0861; ABclonal), p‐LDHA Y10 (AP0889; ABclonal), Gpr132 (DF4894; Affinity Biosciences), LC3 (3868; Cell Signaling Technology), MRC1 (ab300621; Abcam), and MCT4 (22787‐1‐AP; Proteintech) were commercially purchased. Mouse and rabbit Control IgG (AC011 and AC005) was from ABclonal. The MCT1 K473 tri‐methylation rabbit antibody, the peptides of MCT1 K473 non‐, mono‐, di‐, and tri‐methylation were generated by ABclonal, China (https://abclonal.com.cn). The secondary antibodies used for western blot assays were as followed: HRP Goat anti‐Rabbit IgG (H+L) (AS014; ABclonal), HRP Goat anti‐Mouse IgG (H+L) (AS003; ABclonal), HRP‐conjugated AffiniPure Mouse Anti‐Rabbit IgG light chain (AS061; ABclonal), and HRP‐conjugated AffiniPure Goat Anti‐Mouse IgG light chain (AS062; ABclonal). The secondary antibodies applied in immunofluorescence assays were as followed: Dylight 488, Goat Anti‐Rabbit IgG (A23220; Abbkine) and Dylight 549, Goat Anti‐Mouse IgG (A23310; Abbkine).

### Plasmids

The Flag‐SETDB1 and Flag‐SETDB1 H1224K plasmids have been described previously^[^ ^21^ ^]^. The plasmid HA‐MCT1 was purchased from GENECHEM, and the DNAs of MCT1_1‐443_ and MCT1_444‐500_ were cloned into the GV141 vector (GENECHEM). Subsequently, the MCT1 mutants (K462R, K473R, K479R) were generated from the HA‐MCT1 plasmid by a site‐directed mutagenesis kit (C214‐01; Vazyme). The genes of MCT4, P62, Nix, NDP52, Tollip, OPTN, TAX1BP1 were inserted into pcDNA3.1(+) vector. The lentiviral plasmids pLVX‐MCT1‐puro and pLVX‐SETDB1‐neo were constructed by cloning MCT1 and SETDB1 genes into the pLVX‐puro/neo vector. The lentiviral plasmids pLV2‐U6‐SETDB1‐puro or pLV2‐U6‐MCT1‐puro were constructed by inserting the SETDB1 shRNA or the MCT1 shRNA into the pLV2‐U6‐puro vector. The shRNA sequences against human SETDB1 were: 5′‐GCTCAGATGATAACTTCTGTA‐3′, 5′‐AGTTAGAGACATGGGTAATAC‐3′, the sequences against mouse SETDB1 were: 5′‐GAGACTTCATAGAGGAATATA‐3′, 5′‐AAGCAGTTCTCAAGATCTACA‐3′, and the sequence against mouse MCT1 3′UTR was 5′‐GCTTTGTCAGACATTGTTACT‐3′.

### Stable Knockdown and Overexpressing Cell Lines

Stable knockdown cell lines were constructed using lentiviral transduction, and the lentiviruses were generated by co‐transfecting HEK293T cells with plasmids (pLV2‐U6‐SETDB1‐puro, PMD2.G, and psPAX2). In the same way, lentiviruses for the overexpression MCT1 and SETDB1 were obtained. The cells were infected and then treated with 2.0 µg mL^−1^ puromycin for 48 h or 0.8 mg mL^−1^ G418 for 20 days.

### Mass Spectrometry Analyses

HEK293T cells expressing HA‐MCT1 or vector were lysed by NP‐40, followed by IP using Anti‐HA magnetic beads. HA‐MCT1 and associated proteins eluted by HA peptides (100 µg mL^−1^) were loaded to an sodium dodecyl sulfate‐polyacrylamide gel electrophoresis (SDS‐PAGE) gel, and stained by Coomassie brilliant blue.

Mass spectrometry analyses were performed as described previously.^[^ ^50^ ^]^ Briefly, the gels then were sent to the Protein Chemistry and Proteomics Facility of Tsinghua University Technology Center for Protein Research. 0.1 m ammonium acetate in 100% methanol was used to precipitate eluted proteins. After a reduction‐alkylation step (dithiothreitol 5 × 10^−3^ m, iodoacetamide 10 × 10^−3^ m), the samples were followed by sequencing‐grade trypsin digestion overnight. Peptide mixtures were vacuum‐dried in a SpeedVac concentrator and re‐suspended in water containing 0.1% FA (solvent A) before liquid chromatography with tandem mass spectrometry analysis.

The mixtures then were separated using a Thermo Dionex Ultimate 3000 HPLC system directly interfaced with an Orbitrap Q Exactive mass spectrometer (Thermo Fisher Scientific, Bremen, Germany), which was set in the data‐dependent acquisition mode using Xcalibur 2.2 software.

### Immunoblot, Immunoprecipitation, and Immunofluorescence Analysis

For immunoblot assay, CRC tissues and cells were lysed with NP40 buffer containing protease inhibitors and phosphatase inhibitors. Protein quantification was performed by the BCA Protein Assay Kit (23225, Thermo Fisher Scientific). After incubated 10 min at 95 °C, equal amounts of protein were separated by a 10% SDS‐PAGE gel and then transferred to polyvinylidene fluoride (PVDF) membranes (Millipore). After being blocked by 5% skim milk for 1 h, the membranes were incubated with specific antibodies overnight at 4 °C. Subsequently, the membranes were incubated at second antibodies for 1 h and followed by enhanced chemiluminescence detection. For IP, whole‐cell lysates were incubated and rotated overnight with anti‐Flag, anti‐HA beads, or Protein A/G beads conjugated with specific antibodies. Beads were washed four times with lysis buffer and followed by immunoblot assays. For IF staining, the indicated cells were cultured on round cell slides and fixed with 4% paraformaldehyde for 20 min. After permeabilized with 0.3% Triton X‐100, the samples were blocked with 2% bovine serum albumin, and then stained with specific antibodies overnight at 4 °C. Subsequently, the samples were incubated with corresponding fluorescently labeled secondary antibodies (Dylight 488, Goat Anti‐Rabbit IgG, and Dylight 549, Goat Anti‐Mouse IgG) for 2 h at room temperature, and followed by staining with 4,6‐diamidino‐2‐phenylindole. When analyzing tumor samples, the specimens were fixed with 4% paraformaldehyde before staining with anti‐CD206 and anti‐F4/80 antibodies. After that, they were incubated with secondary antibodies. The images were obtained by a confocal fluorescence microscope (Olympus) to assess colocalization.

### In Vitro Binding Assays and In Vitro Methylation Assays

The purified MCT1_444‐500_ protein with N‐terminal His tag (RPE431Hu01) was obtained from Cloud‐Clone Corp, and recombinant SETDB1 protein with N‐terminal Flag tag (31452) was purchased from Active Motif. In vitro binding assays, purified MCT1_444‐500_ protein was incubated with Flag‐SETDB1 fusion protein and then rotated with anti‐Flag magnetic beads overnight at 4 °C. Beads were washed four times with lysis buffer and followed by immunoblot assays. In vitro methylation assays were performed as described elsewhere. Briefly, 1 µg Flag‐SETDB1, 5 µL of 5×PKMT buffer (10 × 10^−3^ m Tris–HCl (pH 8), 2% glycerol, 0.8 × 10^−3^ m KCl, 1 × 10^−3^ m MgCl_2_), 13 × 10^−6^ m S‐adenosyl‐L‐methionine (SAM), purified MCT1_444‐500_ protein as substrates and H_2_O were added to a final volume of 25 µL. The mixtures were incubated at 37 °C for 10 h, and 5×protein sample buffer (250 × 10^−3^ m Tris–HCl (pH 6.8), 10% SDS, 30% glycerol, 5% β‐mercaptoethanol, bromophenol blue) was added to stop the reaction. The mixtures were subjected to SDS‐PAGE, followed by immunoblotting with antibodies.

### Quantitative Real‐Time Polymerase Chain Reaction (qRT‐PCR)

qRT‐PCR assay was performed as previously described.^[^ ^50^ ^]^ The primers used for qPCR are listed in Table S3 in the Supporting Information.

### Membrane and Cytoplasmic Protein Extraction

Cells were cultured and treated in a 10 cm culture dish, and collected by centrifugation (600 *g*, 5 min). The membrane and cytoplasmic protein of the collected cells were further separated using ExKine Membrane and Cytoplasmic Protein Extraction kit (KTP3005, Abbkine), according to the manufacturer's protocols.

### Generation Knockout Cell Lines by the CRISPR/Cas9 System

*MCT1*, *ATG5*, *Beclin 1*, and *Tollip* KO cells were generated by the CRISPR/Cas9 system. The single guide RNAs were designed by a public web, E‐CRISP (http://www.e‐crisp.org/E‐CRISP/), and cloned into the PX462 plasmid (62987, addgene) or PX459 plasmid (62988, addgene). After the plasmids were transfected into cells for 24 h, cells were selected with puromycin for 48 h. The single cell was cultured in a 96‐well plate for 2 weeks and then transferred to 12 well plates. The KO cells were validated by immunoblot and sanger sequencing. The sgRNA sequences were as follows:

Human *MCT1* sgRNA: #1 5′‐GACTTCCATATTTATTCACC‐3′; #2 5′‐GTGGCTGCTTGTCAGGCTG‐3′;

Human *ATG5* sgRNA: 5′‐GTGCTTCGAGATGTGTGGTT‐3′;

Human *Beclin 1* sgRNA: 5′‐ATTTATTGAAACTCCTCGCC‐3′;

Human *Tollip* sgRNA: 5′‐GCTGCAGTACGGAGGCGCAG‐3′.

### Glucose Uptake and Lactate Production Assay

For glucose uptake assay, cells were plated on 96‐well culture dishes and cultured with a complete medium for 24 h. After removing the complete medium, cells were cultured with a low glucose medium for 4 h. Next, the ability of cells to take up glucose was measured using Glucose Uptake Assay Kit (TV785, Dojindo). For lactate production assay, cells were plated on 6‐well culture dishes and cultured with a complete medium for 24 h. The culture medium was changed to a fresh medium and incubated for another 6 h. The lactate content in the cultured medium was measured by L‐Lactate Assay Kit (Colorimetric) (ab65331, Abcam). Glucose uptake and lactate production were normalized by the concentration of protein.

### Bioenergetic Analysis

For detection of glycolytic activity, the Glycolysis‐Stress Test Kit (103020‐100, Agilent Technologies) was used. The indicated cells were plated at an XF24 plate and cultured in an incubator without CO_2_ overnight. Then, the medium was exchanged with glucose‐free buffered Seahorse media (pH 7.4) for 1 h. Glucose, oligomycin, and 2‐DG were loaded into sensor ports to achieve final concentrations of 10 × 10^−3^, 1 × 10^−6^, and 50 × 10^−3^ m, respectively. The XF24 plate was placed into the Seahorse XF24 analyzer (Seahorse Bioscience, North Billerica, MA, USA), and the real‐time extracellular acidification rate (ECAR) was measured. Data were normalized by the concentration of protein in each well.

### Tumor Supernatant Preparation and Collection

Cell lines were cultured at normal conditions. Fractionation of CRC tumor supernatants was obtained using Amico Ultra centrifugal filters (3K Ultracel, Millipore). The supernatant fraction >3 kDa remained above the filter, and the fraction <3 kDa passed through to the lower chamber.

### Isolation of BMDMs

Isolation of BMDMs was performed as described elsewhere.^[^ ^6^ ^]^ BMDMs were harvested from bone‐marrow precursor cells by lavaging the tibias and femurs of 6 weeks old BALB/c mice. After filtering and washing, 1 mL ACK lysing buffer was added into the tubes for 1 min, and then 10 mL RPMI‐1640 was added to neutralize. After centrifugation, cells were plated at 12‐well plates and cultured with RPMI‐1640 medium containing 50 ng mL^−1^ M‐CSF (HY‐P7085, MedChemExpress) for 7 days. Then, BMDMs were stimulated with the indicated conditioned medium, followed by qPCR and IB assay.

### Syngeneic Tumor Model

All animal experiments were approved by the Animal Care and Use Committee of Tongji Hospital. The BALB/c mice were purchased from Vital River Laboratory Animal Technologies (Beijing, China). The indicated CT26 cells (2 × 10^5^) were injected subcutaneously into 6 weeks old female BALB/c mice. Tumor volumes were measured at the indicated time, followed by calculating using the formula: 0.5 × *L* × *D*2 (*L*: major axis, *D*: minor axis). After the mice were sacrificed, the subcutaneous tumors were weighed, and the tissues were further investigated.

### Preparation of Single‐Cell Suspensions from Subcutaneous Tumors

The mouse subcutaneous tumors were cut into pieces with scissors, and digested with 3 mL serum‐free RPMI‐1640 medium containing collagenase IV (50 µL 25 mg mL^−1^; V900893, Sigma‐Aldrich), Hyaluronidase (50 µL 32 mg mL^−1^, H3506, Sigma‐Aldrich), and DNase I (25 µL 10 mg mL^−1^; 10104159001, Roche) for 1 h in a 37 °C shaking incubator (150 r.p.m.). After full enzymatic dissociation, 7 mL serum‐free RPMI‐1640 medium was added into tubes to dilute enzyme concentration. After filtration and centrifugation, 1 mL ACK lysing buffer was added into the tubes for 1 min to lyse red blood cells, followed by neutralization. The samples were resuspended and then kept on ice during the following staining experiment.

### Flow Cytometry Analysis

The BMDMs or single cell suspensions from subcutaneous tumors were blocked by anti‐mouse CD16/32 antibody (101319, BioLegend) at a 1:200 dilution for 30 min. After washing and centrifugation, the samples were stained with Zombie UV Fixable Viability Kit (423106, BioLegend) to exclude dead cells. Then, the samples were stained with anti‐CD45‐PerCP antibody (103129, BioLegend), anti‐CD11b‐PE antibody (101207, BioLegend), anti‐F4/80‐APC antibody (123115, BioLegend), anti‐CD86‐FITC antibody (105005, BioLegend), and anti‐CD206‐PE/Cyanine7 antibody (141719, BioLegend) at 25 °C for 30 min in the dark. The samples were then washed twice and resuspended in phosphate‐buffered saline buffer. The labeled cells were analyzed by flow cytometry, and the results were analyzed by FlowJo software.

### Immunohistochemistry

IHC was performed on tissues from subcutaneous tumors and tissue microarrays. Briefly, after deparaffinization, hydration, and antigen retrieval, the tissue sections were incubated with a blocking buffer for 30 min at room temperature. Next, the sections were stained with the indicated primary antibodies overnight at 4 °C, followed by incubation with the second antibodies. For MCT1 K473 tri‐methylation and SETDB1 staining scores, the intensity and density of positive cells were used to calculate the IHC scores. The intensity of positive cells was classified as s 0 (no staining), 1 (weak), 2 (moderate), and 3 (strong), and the density of positive cells was divided into four levels: 1 (< 5%), 2 (5−30%), 3 (30−70%), and 4 (staining >70%). Then, the total IHC scores were generated by multiplying these two scores (0 to 12). According to the scores, the score of cases from 0 to 6 was regarded as the low expression group, whereas the score of cases from 7 to 12 was assigned to the high expression group. The tissue microarrays were evaluated by two independent pathologists. For CD206 staining scores, we randomly selected three representative fields per tissue section and examined them under a microscope at 400x magnification. The average number of positively stained cells in these fields was used as a representation for each case.

### Statistical Analysis

All statistical analyses were performed using GraphPad 8.0 and Origin 2022. The Student's two‐tailed *t*‐test, one‐way ANOVA with Tukey's test, or two‐way ANOVA with Tukey's test was used to determine the statistical significance of differences between groups. The chi‐square test was applied to categorical variables. The spearman test was performed for correlation analysis. The Kaplan–Meier method and log‐rank test were utilized to calculate overall survival. *p* < 0.05 indicated the difference was statistically significant. *p < 0.05, **p < 0.01, ***p < 0.001, ****p < 0.0001.

## Conflict of Interest

The authors declare no conflict of interest.

## Author Contributions

X.S., F.X., and L.S. conceived and designed the experiments; X.S., Q.W., and Z.R. conducted biochemical and cellular experiments. D.S., C.H., and S.F. performed the animal experiments. A.L., L.L., K.W., and X.L. generated the gene knock‐out cell lines. C.Y. and C.Q. provided help for bioinformatics analysis. X.L., J.H., and G.W. gave suggestions for many experiments. X.S., F.X., and L.S. organized and analyzed the data and wrote the manuscript, which was edited by all authors.

## Supporting information

Supporting Information

Supplemental Table 1

Supplemental Table 2

Supplemental Table 3

## Data Availability

The data that support the findings of this study are available from the corresponding author upon reasonable request.
