# Supplementary material for: SETDB1 Methylates MCT1 Promoting Tumor Progression by Enhancing the Lactate Shuttle
Source: Adv Sci (Weinh). 2023 Aug 4;10(28):2301871. doi: 10.1002/advs.202301871 (PMC10558670; doi:10.1002/advs.202301871)
Supplement: Supplementary file 1 — Supporting Information [file ADVS-10-2301871-s001.pdf]

ADVANCED SCIENCE
Open Access

Supporting Information

for *Adv. Sci.*, DOI 10.1002/advs.202301871

SETDB1 Methylates MCT1 Promoting Tumor Progression by Enhancing the Lactate Shuttle

Xiaowei She, Qi Wu, Zejun Rao, Da Song, Changsheng Huang, Shengjie Feng, Anyi Liu, Lang Liu, Kairui Wan, Xun Li, Chengxin Yu, Cheng Qiu, Xuelai Luo, Junbo Hu, Guihua Wang, Feng Xu and Li Sun**

SETDB1 Methylates MCT1 Promoting Tumor Progression by Enhancing the Lactate Shuttle

Xiaowei She[1], Qi Wu[1], Zejun Rao[1], Da Song[1], Changsheng Huang[1], Shengjie Feng[1], Anyi Liu[1], Lang Liu[1], Kairui Wan[1], Xun Li[1], Chengxin Yu[1], Cheng Qiu[1], Xuelai Luo[1], Junbo Hu[1], Guihua Wang[1,2], Feng Xu[1,*], Li Sun[3,*]

[1]GI Cancer Research Institute, Tongji Hospital, Huazhong University of Science and Technology, Wuhan, 430030, P. R. China

[2]Tongji Medical College and State Key Laboratory for Diagnosis and Treatment of Severe Zoonotic Infectious Disease, Huazhong University of Science and Technology, Wuhan, Hubei, 430030, P. R. China

[3]Department of Oncology, Tongji Hospital, Huazhong University of Science and Technology, Wuhan, 430030, P. R. China

*** To whom all correspondence should be sent:**

Li Sun, Department of Oncology, Tongji Hospital, Huazhong University of Science and Technology, Wuhan, 430030, P. R. China. E-mail: litchisun@tjh.tjmu.edu.cn.

Feng Xu, GI Cancer Research Institute, Tongji Hospital, Huazhong University of Science and Technology, Wuhan, 430030, P. R. China. E-mail: fxu331@tjh.tjmu.edu.cn.

Key Words: Lactate shuttle; MCT1 methylation; SETDB1; Tumor progression

Running title: SETDB1 methylates MCT1 enhancing lactate shuttle.

Conflict-of-interest disclosure: Authors declare no competing financial interests.

Supplementary figure legend

Figure S1.

SETDB1 enhances the expression of MCT1. a) Mass spectrometry analysis shows sequences of identified unique peptides corresponding to SETDB1. A full list of identified MCT1 interactors is shown in Table S1, Supporting Information. The mass spectrometry was performed once. b) HEK293T cells were co-transfected with Flag-SETDB1 and Vector or HA-MCT1 (WT, 1-443aa and 444-500aa) plasmids, followed by IP assays. c,d) Protein and mRNA expression of MCT1 were measured by IB assays and qRT-PCR assays in HT29 cells transfected with Vector, Flag-SETDB1 (WT), or Flag-SETDB1 (H1224K) plasmids. e,f) Protein and mRNA expression of MCT1 were determined by IB assays and qRT-PCR assays in HT29 cells treated with Mithramycin A at indicated concentrations. g) The membrane and cytosolic fractions from SW480 cells treated with Mithramycin A were collected and subjected to IB analysis. h) The correlation between the mRNA expression of MCT1 and SETDB1 was determined by the Pearson correlation coefficient test, p = 0.343, r = 0.037. Colon adenocarcinoma (COAD), and Rectum adenocarcinoma (READ). All immunoblots were performed three times, independently, with similar results. d,f) Data are represented as mean ± s.d. ns means no significant, by one-way ANOVA with Tukey's test.

Figure S2.

SETDB1 represses the autophagic degradation of MCT1. a) The degradation of MCT1 was detected in shSETDB1NC and shSETDB1#1 HT29 cells treated with CHX (100 μg/ml) for 0, 4, 8, or 12 h by CHX-chase assay. b) Quantification of the relative protein level of MCT1 in (a). c) The degradation of MCT1 was measured in SW480 cells treated with DMSO or Mithramycin A (100×10^{-9} M, 24 h) upon starvation-induced autophagy activation under Earle's balanced salt solution (EBSS)-cultured condition for 0, 4, 8, or 12 h by IB assays. d) WCL from WT and *ATG5* or *Beclin 1* KO SW480 cells were collected for IB analysis. e) SW480 cells transfected with Vector or Flag-SETDB1, WCL were collected for IP with anti-MCT1 antibody, followed by IB analysis. f) WCL collected from SETDB1 knockdown SW480 were subjected to IP

assay with anti-MCT1 antibody, followed by IB analysis. All immunoblots were performed three times, independently, with similar results. b) Data are represented as mean ± s.d. ***$p < 0.001$, by two-way ANOVA with Tukey's test.

Figure S3.

MCT1 is methylated by SETDB1 at Lys 473. a) HEK293T cells transfected with HA-MCT1(left) or HA-MCT4 (right) plasmid, then transfected with Vector or Flag-SETDB1. WCL were collected for IP with anti-HA beads, followed by IB analysis. b) WCL collected from SW480 and HT29 cells treated by Mithramycin A at indicated concentrations were subjected to IP assay with anti-MCT1 antibody, followed by IB analysis. c) Showing results from mass spectrometry analysis of MCT1 potential methylation sites. d) The MCT1 K473 site amino acid in different species. e) Sequencing of *MCT1* knockout alleles in SW480 cells after CRISPR-Cas9 mediated editing and gRNA designs. f) WCL from *MCT1* WT and *MCT1* KO SW480 cells were collected for IB analysis. g) Representative image of IHC staining for MCT1 K473 tri-methylation in the indicated tumor tissues. Black scale bar, 50 μm. h) MCT methylation was detected by MCT1 K473 specific tri-methylation antibody in SW480 cells transfected with Vector, Flag-SETDB1 (WT), or Flag-SETDB1 (H1224K) plasmids. i) SW480$^{MCT1\ \mathrm{KO}}$ cells stably expressing MCT1 WT or MCT1 K473R were transfected with Vector or Flag-SETDB1, and WCL were collected for IB assays. All immunoblots were performed three times, independently, with similar results.

Figure S4.

SETDB1-mediated MCT1 methylation inhibits the interaction between MCT1 and Tollip. a) The degradation of MCT1 WT or K473R mutant in SW480 cells was measured by CHX-chase assay in the presence of inhibitors MG132 (10×10^{-6} M), CQ (50×10^{-6} M), or 3-MA (5×10^{-3} M). b) Quantification of the relative protein level of MCT1 in (a). c) HEK293T cells were co-transfected with HA-Ub and Vector, Flag-MCT1 WT or Flag-MCT1 K473R, and WCL were collected for IP with anti-Flag beads, followed by IB analysis. d) WCL from *Tollip* WT and *Tollip* KO SW480 cells were

collected for IB analysis. e) SW480$^{MCT1\ KO}$ cells transfected with HA-MCT1 WT or K473R plasmid as indicated, WCL were collected for IP with anti-HA beads, followed by IB analysis. f) IF analysis of Tollip (green) and MCT1 (red) in SW480 cells with or without Mithramycin A (100 × 10^{-9} M, 24 h) treatment, or overexpression of SETDB1, followed by EBSS treatment together with Bafilomycin A1 (0.2 × 10^{-6} M). The nucleus is stained with DAPI. White scale bars, 5 µm. g) Statistics of colocalization of MCT1 and Tollip indicated by the Pearson's correlation. All immunoblots were performed three times, independently, with similar results. g) Data are represented as mean ± s.d. *$p < 0.05$, *$p < 0.01$, by one-way ANOVA with Tukey's test.

Figure S5.

SETDB1 facilitates tumor glycolysis and M2-like polarization of TAMs by regulating lactate transport. a,b) Lactate production was measured in the CM of SW480 and HT29 cells silenced with control (shNC) or SETDB1 shRNA (#1, #2), n=3. c,d) Glucose uptake was measured in SW480 and HT29 cells silenced with control (shNC) or SETDB1 shRNA (#1, #2), n=3. e,f) Bioenergetic analysis was performed with the Seahorse XF24 analyzer platform. ECAR of SW480 cells silenced with control (shNC) or SETDB1 shRNA (#1, #2) was measured and calculated, n=3. g) MCT1 3'UTR knockdown CT26 cell lines were successfully established by lentivirus containing shRNA against MCT1 3'UTR, and rescued with mus-MCT1 WT and mus-MCT1 K467R (up). SETDB1 knock-down CT26 cell lines were successfully established by using lentivirus containing shRNA against SETDB1(down). h,i) Lactate production was measured in the CM of CT26 cells under indicated treatment, n=3. j,k) Glucose uptake was measured in CT26 cells under indicated treatment, n=3. l) A syngeneic tumor model was performed by injecting CT26 cells silenced with control (shNC) or SETDB1 shRNA (#1, #2) into BALB/c mice. n = 5 mice. m,n) Quantification of tumor weight and volume of tumors generated in (l). o,p) Flow cytometry analysis of macrophage polarization of tumors generated in (l). All immunoblots were performed three times, independently, with similar results. Data are represented as mean ± s.d. *$p < 0.05$, **$p < 0.01$, ***$p < 0.001$, ****$p < 0.0001$; ns means no significant, by a-d,h-

k,m,p) one-way ANOVA with Tukey's test and f,n) two-way ANOVA with Tukey's test.

Figure S6.

SETDB1-mediated tumor progression partly depends on MCT1 methylation. a) MCT1-3'UTR CT26 cells stably expressing MCT1 WT or MCT1 K473R cells and overexpression Vector or SETDB1 were successful established. b) WCL collected from BMDMs treated by the indicated CM, followed by IB assay. c) Lactate production was measured in the CM of CT26 cells under indicated treatment, n=3. d) Glucose uptake was measured in CT26 cells under indicated treatment, n=3. e) Representative images for infiltration of intratumor $CD206^+$ and $F4/80^+$ cells by IF. White scale bars, 20 μm. All immunoblots were performed three times, independently, with similar results. c,d) Data are represented as mean ± s.d. ***$p < 0.001$, ****$p < 0.0001$; ns means no significant, by one-way ANOVA with Tukey's test.

Figure S7.

SETDB1 is highly expressed in human cancers. a) SETDB1 mRNA expression was compared using box plots in COAD&READ, Breast invasive carcinoma (BRAC), Liver hepatocellular carcinoma (LIHC), Lung adenocarcinoma (LUAD), Lung squamous cell carcinoma (LUSC), and Stomach adenocarcinoma (STAD). The data were represented as a box plot and the line showed the mean. Student's two-tailed t-test, $p < 0.001$. b,c) Kaplan-Meier analysis of Disease-Specific Survival and Progress Free Interval in TCGA-COAD&READ according to SETDB1 expression. Log-rank test, $p = 0.013$ and $p = 0.003$, respectively. d) Kaplan-Meier analysis of Overall survival in TCGA-STAD according to SETDB1 expression. Log-rank test, $p = 0.004$. e) Scores (0-3) indicate the intensity of SETDB1 and MCT1 K473 tri-methylation positive cells in colorectal tumor tissues. Black scale bar, 50 μm. f) SETDB1 IHC staining score was detected in tumor and adjacent tissues, n=109. Student's two-tailed t-test, $p < 0.0001$. g) Kaplan-Meier analysis of overall survival in a set of 80 colorectal cancer patients according to SETDB1 expression. Log-rank test, $p = 0.0124$.

Figure S1

a **E9PRF4 Histone-lysine N-methyltransferase SETDB1 (Fragment) OS=Homo sapiens** OX=9606 GN=**SETDB1** PE=1 SV=1 - [E9PRF4_HUMAN]

Sequence	Modifications	Start	Stop
ASTSGLGIKDEGDIK	-	1024	1038
QFYDGEEScYIIDAK	C9(Carbamidomethyl)	1199	1213

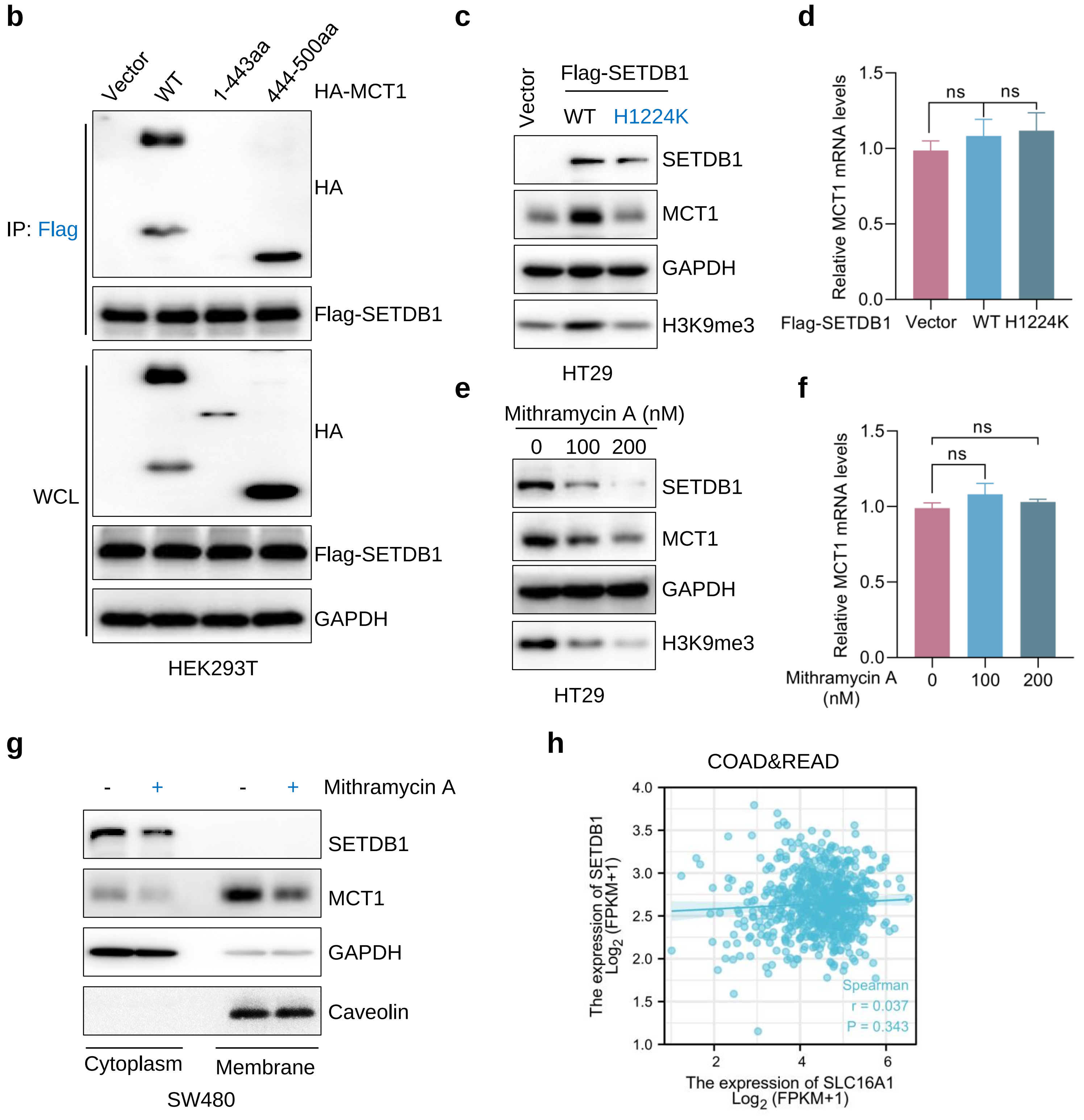

Figure S2

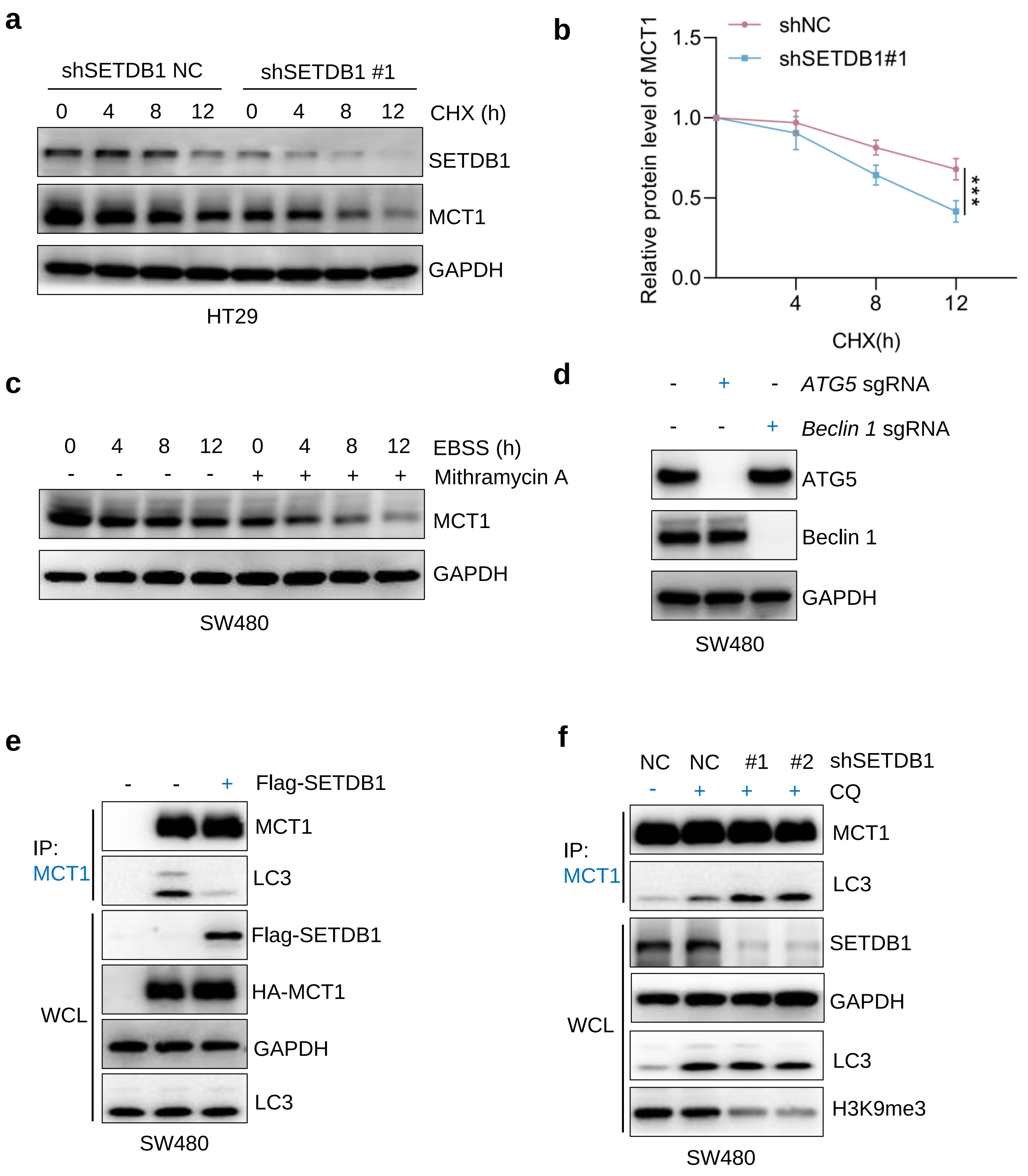
a
shSETDB1 NC
shSETDB1 #1
0 4 8 12 0 4 8 12 CHX (h)
SETDB1
MCT1
GAPDH
HT29
b
shNC
shSETDB1#1
Relative protein level of MCT1
1.5
1.0
0.5
0.0
4
8
12
CHX(h)
***
c
0 4 8 12 0 4 8 12 EBSS (h)
- - - - + + + + Mithramycin A
MCT1
GAPDH
SW480
d
- + - ATG5 sgRNA
- - + Beclin 1 sgRNA
ATG5
Beclin 1
GAPDH
SW480
e
- - + Flag-SETDB1
IP: MCT1
MCT1
LC3
WCL
Flag-SETDB1
HA-MCT1
GAPDH
LC3
SW480
f
NC NC #1 #2 shSETDB1
- + + + CQ
IP: MCT1
MCT1
LC3
WCL
SETDB1
GAPDH
LC3
H3K9me3
SW480

Figure S3

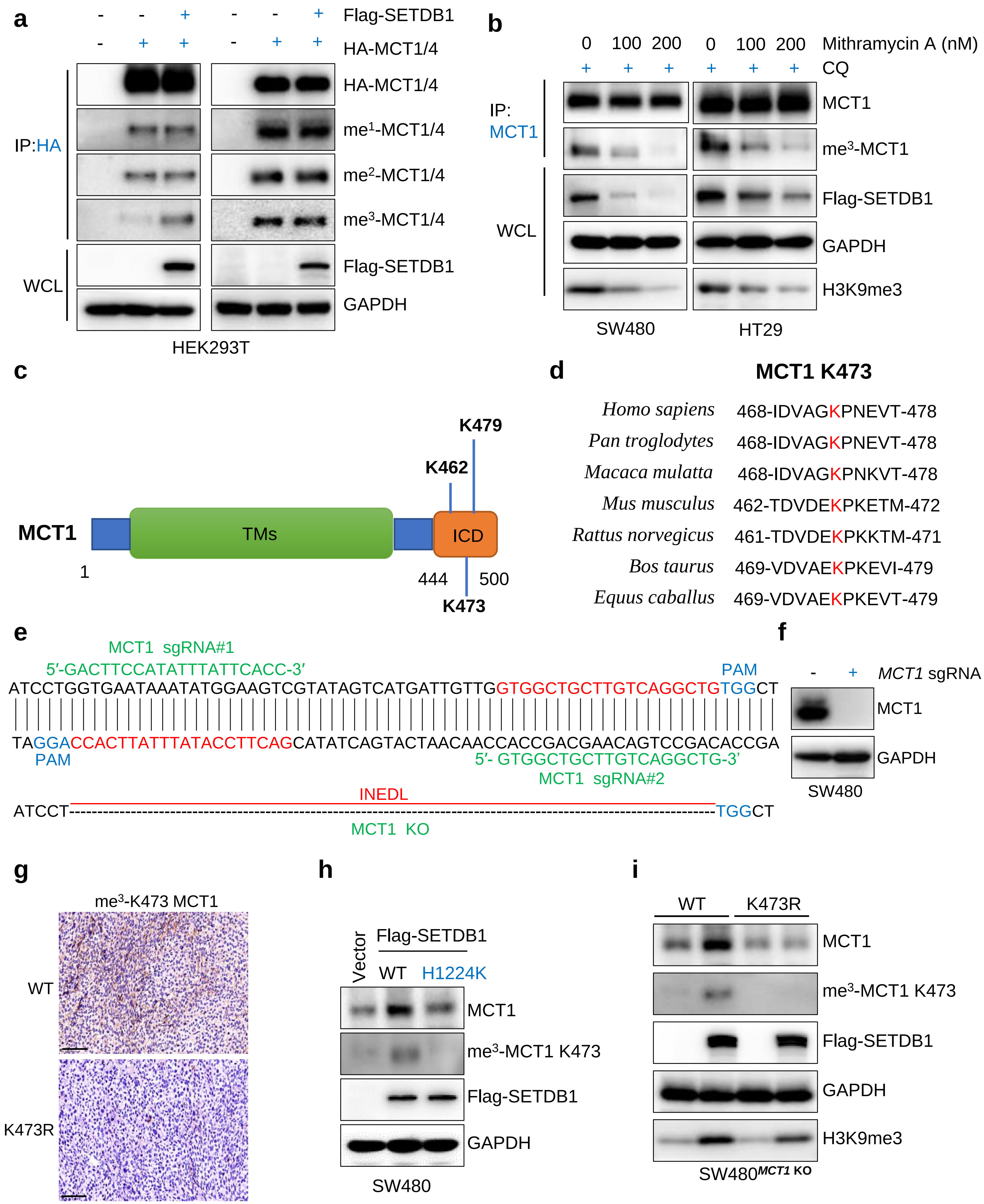
a
Flag-SETDB1
HA-MCT1/4
IP:HA
HA-MCT1/4
me1-MCT1/4
me2-MCT1/4
me3-MCT1/4
WCL
Flag-SETDB1
GAPDH
HEK293T
b
0 100 200 0 100 200 Mithramycin A (nM)
CQ
IP: MCT1
MCT1
me3-MCT1
WCL
Flag-SETDB1
GAPDH
H3K9me3
SW480
HT29
c
K479
K462
MCT1
TMs
ICD
1
444
500
K473
d
MCT1 K473
Homo sapiens 468-IDVAGKPNEVT-478
Pan troglodytes 468-IDVAGKPNEVT-478
Macaca mulatta 468-IDVAGKPNKVT-478
Mus musculus 462-TDVDEKPKETM-472
Rattus norvegicus 461-TDVDEKPKKTM-471
Bos taurus 469-VDVAEKPKEVI-479
Equus caballus 469-VDVAEKPKEVT-479
e
MCT1 sgRNA#1
5′-GACTTCCATATTTATTCACC-3′
PAM
ATCCTGGTGAATAAATATGGAAGTCGTATAGTCATGATTGTTGGTGGCTGCTTGTCAGGCTGTGGCT
TAGGACCACTTATTTATACCTTCAGCATATCAGTACTAACAACCACCGACGAACAGTCCGACACCGA
PAM
5′- GTGGCTGCTTGTCAGGCTG-3′
MCT1 sgRNA#2
INEDL
ATCCT--TGGCT
MCT1 KO
f
- + MCT1 sgRNA
MCT1
GAPDH
SW480
g
me3-K473 MCT1
WT
K473R
h
Vector
Flag-SETDB1
WT H1224K
MCT1
me3-MCT1 K473
Flag-SETDB1
GAPDH
SW480
i
WT K473R
MCT1
me3-MCT1 K473
Flag-SETDB1
GAPDH
H3K9me3
SW480MCT1 KO

Figure S4

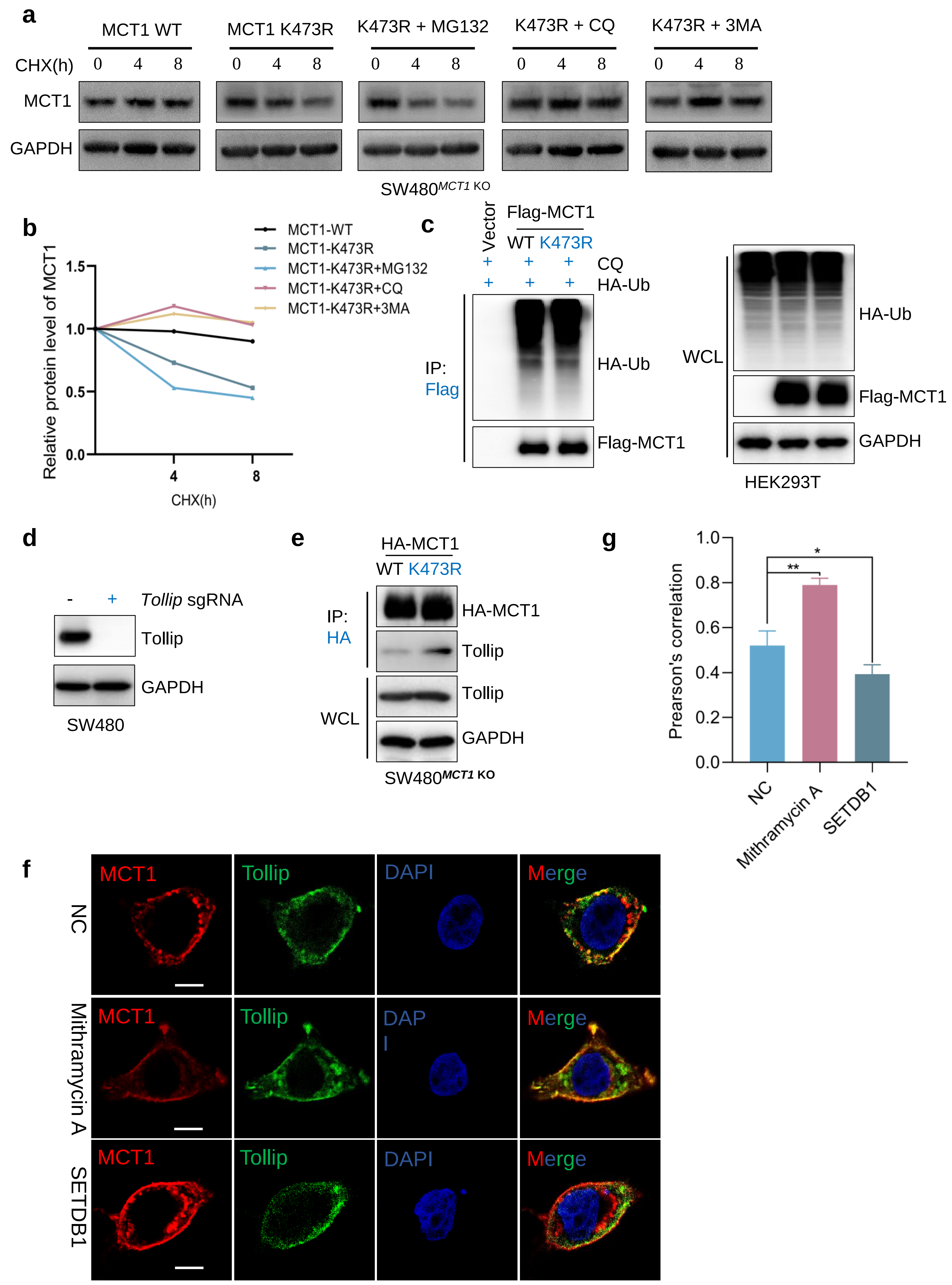
a
MCT1 WT
MCT1 K473R
K473R + MG132
K473R + CQ
K473R + 3MA
CHX(h)
0 4 8
MCT1
GAPDH
SW480MCT1 KO
b
MCT1-WT
MCT1-K473R
MCT1-K473R+MG132
MCT1-K473R+CQ
MCT1-K473R+3MA
Relative protein level of MCT1
1.5
1.0
0.5
0.0
4
8
CHX(h)
c
Vector
Flag-MCT1
WT K473R
CQ
HA-Ub
IP:
Flag
HA-Ub
Flag-MCT1
WCL
HA-Ub
Flag-MCT1
GAPDH
HEK293T
d
- + Tollip sgRNA
Tollip
GAPDH
SW480
e
HA-MCT1
WT K473R
IP:
HA
HA-MCT1
Tollip
WCL
Tollip
GAPDH
SW480MCT1 KO
g
Prearson's correlation
1.0
0.8
0.6
0.4
0.2
0.0
**
*
NC
Mithramycin A
SETDB1
f
MCT1
Tollip
DAPI
Merge
NC
Mithramycin A
SETDB1

Figure S5

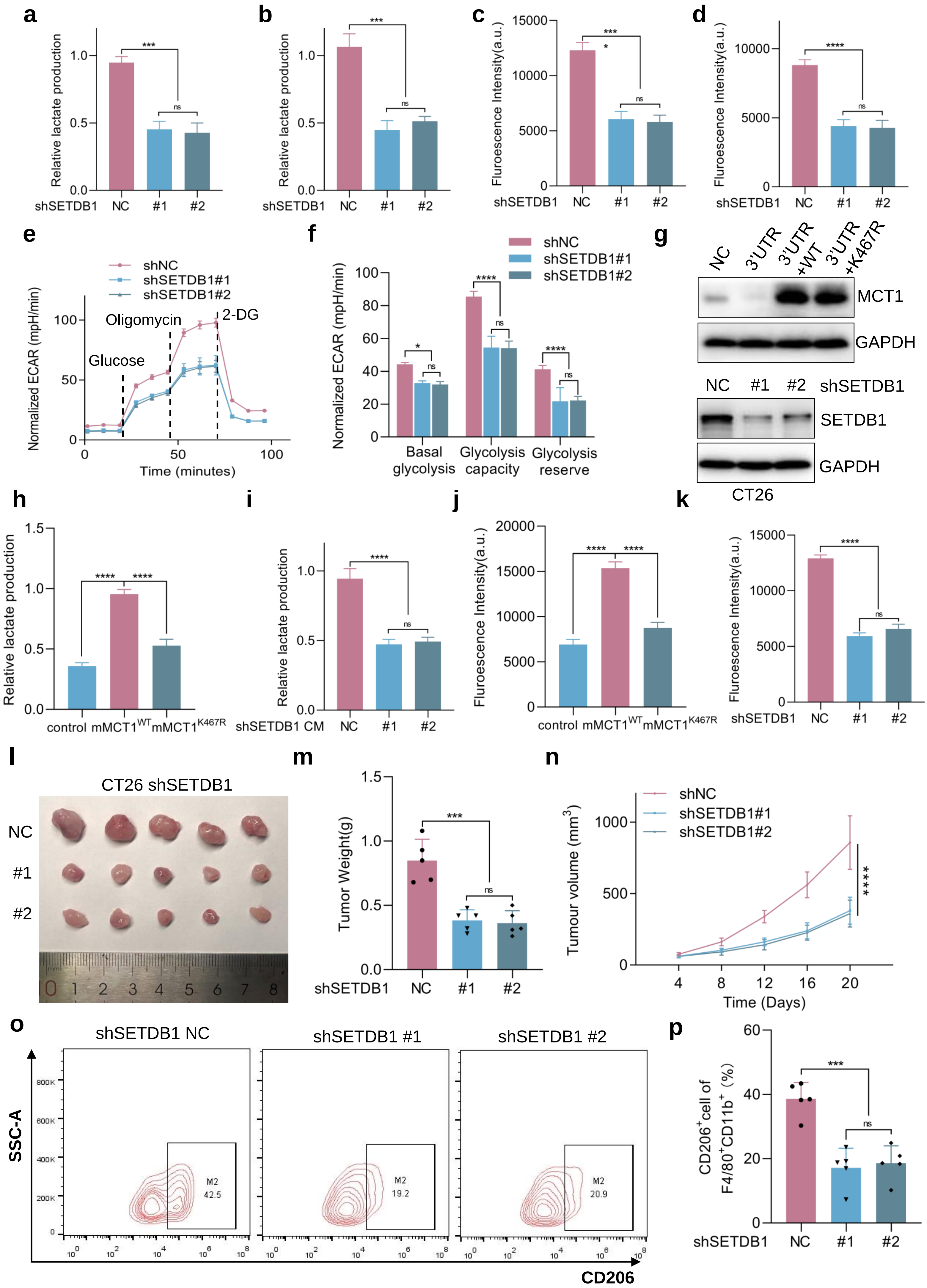

a
Relative lactate production
shSETDB1 NC #1 #2
b
Relative lactate production
shSETDB1 NC #1 #2
c
Fluroescence Intensity(a.u.)
shSETDB1 NC #1 #2
d
Fluroescence Intensity(a.u.)
shSETDB1 NC #1 #2
e
shNC
shSETDB1#1
shSETDB1#2
Oligomycin
2-DG
Glucose
Normalized ECAR (mpH/min)
Time (minutes)
f
shNC
shSETDB1#1
shSETDB1#2
Normalized ECAR (mpH/min)
Basal glycolysis
Glycolysis capacity
Glycolysis reserve
g
NC
3'UTR
3'UTR +WT
3'UTR +K467R
MCT1
GAPDH
NC #1 #2 shSETDB1
SETDB1
GAPDH
CT26
h
Relative lactate production
control mMCT1WT mMCT1K467R
i
Relative lactate production
shSETDB1 CM NC #1 #2
j
Fluroescence Intensity(a.u.)
control mMCT1WT mMCT1K467R
k
Fluroescence Intensity(a.u.)
shSETDB1 NC #1 #2
l
CT26 shSETDB1
NC
#1
#2
m
Tumor Weight(g)
shSETDB1 NC #1 #2
n
shNC
shSETDB1#1
shSETDB1#2
Tumour volume (mm3)
Time (Days)
o
shSETDB1 NC
shSETDB1 #1
shSETDB1 #2
SSC-A
CD206
M2 42.5
M2 19.2
M2 20.9
p
CD206+cell of F4/80+CD11b+ (%)
shSETDB1 NC #1 #2

Figure S6

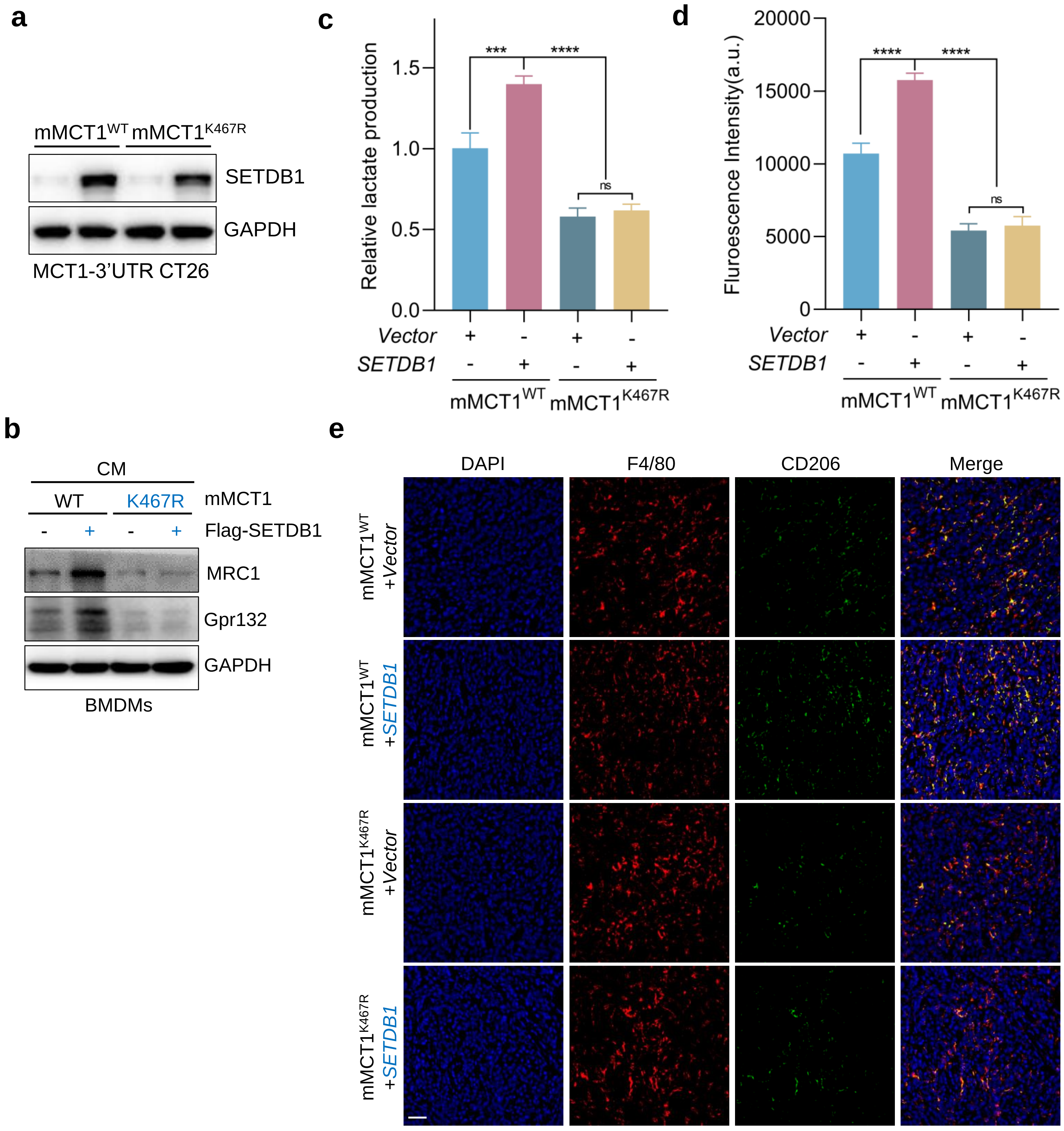
a
mMCT1WT mMCT1K467R
SETDB1
GAPDH
MCT1-3'UTR CT26
c
Relative lactate production
1.5
1.0
0.5
0.0
***
****
ns
Vector
SETDB1
mMCT1WT
mMCT1K467R
d
Fluroescence Intensity(a.u.)
20000
15000
10000
5000
0
****
****
ns
Vector
SETDB1
mMCT1WT
mMCT1K467R
b
CM
WT
K467R
mMCT1
Flag-SETDB1
MRC1
Gpr132
GAPDH
BMDMs
e
DAPI
F4/80
CD206
Merge
mMCT1WT +Vector
mMCT1WT +SETDB1
mMCT1K467R +Vector
mMCT1K467R +SETDB1

Figure S7

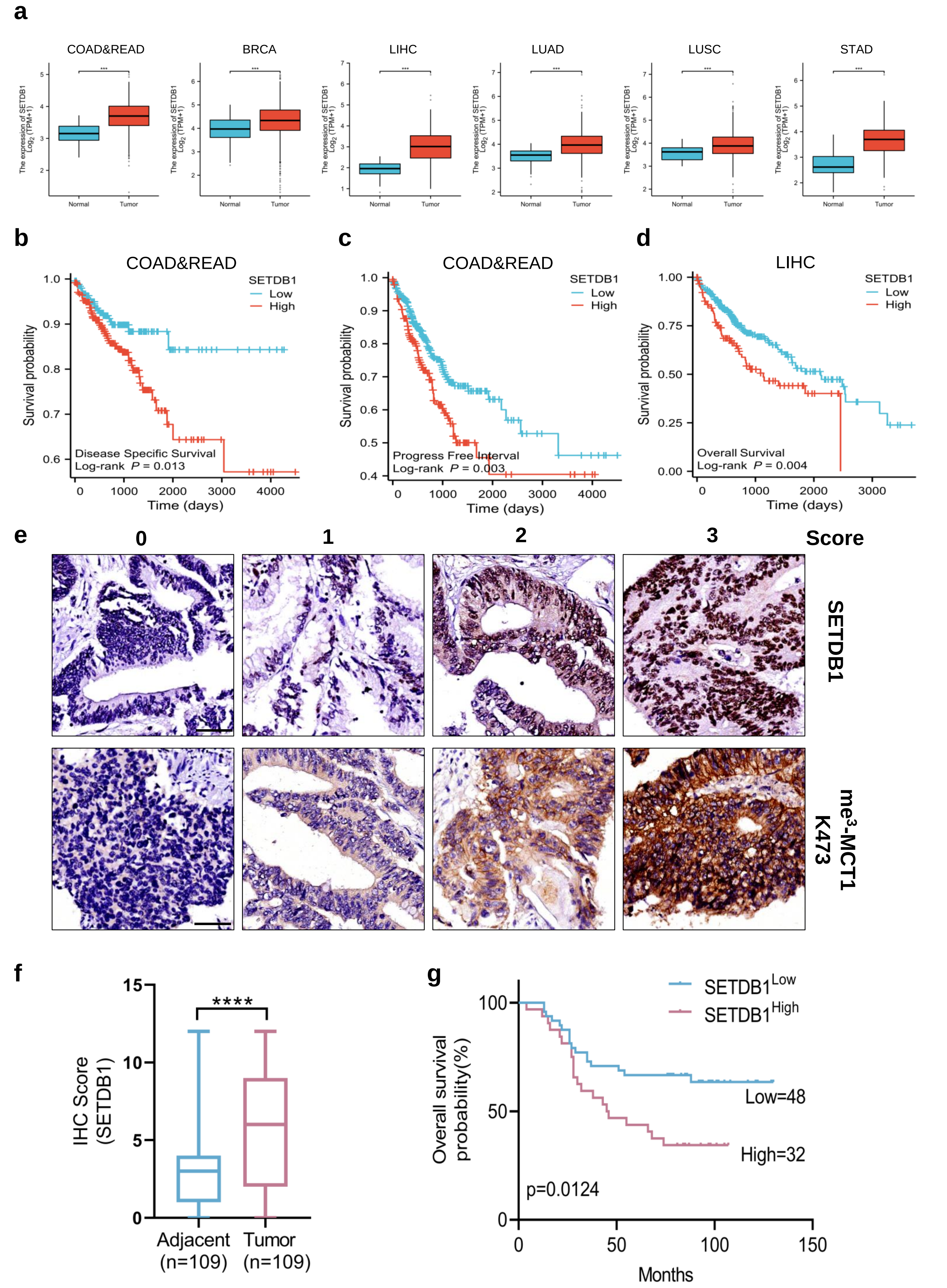
a
COAD&READ
BRCA
LIHC
LUAD
LUSC
STAD
The expression of SETDB1 Log2 (TPM+1)
Normal
Tumor
b
COAD&READ
SETDB1
Low
High
Survival probability
Disease Specific Survival
Log-rank P = 0.013
Time (days)
c
COAD&READ
Progress Free Interval
Log-rank P = 0.003
d
LIHC
Overall Survival
Log-rank P = 0.004
e
0
1
2
3
Score
SETDB1
me3-MCT1 K473
f
****
IHC Score (SETDB1)
Adjacent (n=109)
Tumor (n=109)
g
SETDB1Low
SETDB1High
Overall survival probability(%)
Low=48
High=32
p=0.0124
Months
